# Advances in Intracellular Calcium Signaling Reveal Untapped Targets for Cancer Therapy

**DOI:** 10.3390/biomedicines9091077

**Published:** 2021-08-24

**Authors:** Aarushi Sharma, Grace T. Ramena, Randolph C. Elble

**Affiliations:** 1 Department of Pharmacology and Simmons Cancer Institute, Southern Illinois University School of Medicine, Springfield, IL 62702, USA; sharma.aarushi1821@gmail.com; 2 Department of Aquaculture, University of Arkansas, Pine Bluff, AR 71601, USA; ramenag@uapb.edu

**Keywords:** SOCE, ORAI, STIM, STIMATE, SERCA, PNCA, calmodulin, TRP, IP3R, MCU, VGCC

## Abstract

Intracellular Ca^2+^ distribution is a tightly regulated process. Numerous Ca^2+^ chelating, storage, and transport mechanisms are required to maintain normal cellular physiology. Ca^2+^-binding proteins, mainly calmodulin and calbindins, sequester free intracellular Ca^2+^ ions and apportion or transport them to signaling hubs needing the cations. Ca^2+^ channels, ATP-driven pumps, and exchangers assist the binding proteins in transferring the ions to and from appropriate cellular compartments. Some, such as the endoplasmic reticulum, mitochondria, and lysosomes, act as Ca^2+^ repositories. Cellular Ca^2+^ homeostasis is inefficient without the active contribution of these organelles. Moreover, certain key cellular processes also rely on inter-organellar Ca^2+^ signaling. This review attempts to encapsulate the structure, function, and regulation of major intracellular Ca^2+^ buffers, sensors, channels, and signaling molecules before highlighting how cancer cells manipulate them to survive and thrive. The spotlight is then shifted to the slow pace of translating such research findings into anticancer therapeutics. We use the PubMed database to highlight current clinical studies that target intracellular Ca^2+^ signaling. Drug repurposing and improving the delivery of small molecule therapeutics are further discussed as promising strategies for speeding therapeutic development in this area.

## 1. Introduction

**Figure 1:**
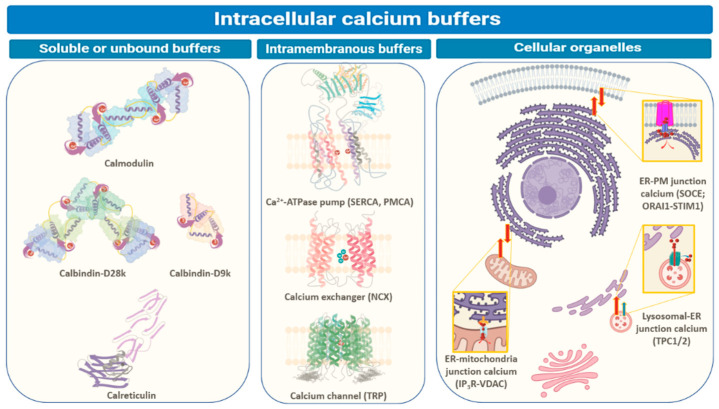
Types of intracellular Ca^2+^ buffers.

Ca^2+^ is the quintessential ion central to numerous cellular homeostasis and physiological functions. Low hydration energy, high polarizability, relative flexibility of coordination sites and bond length, and large concentration gradient across cellular membranes (100 nM intracellular to 2 mM extracellular) due to low intracellular levels make it the ion of choice at the core of cellular signaling in prokaryotes and eukaryotes alike [1,2]. The mechanisms adopted by cells for intracellular Ca^2+^ buffering involve sequestration by special proteins [3,4] (Figure 1). Some of these proteins exist in the soluble or non-membranous parts of the cytoplasm within or outside organelles that serve as repositories for Ca^2+^ ions [3]. Such proteins sequester cytosolic Ca^2+^ upon sensing an increase in its levels and participate in relaying the associated cellular messages. Other proteins that work as intracellular Ca^2+^ buffers exist in the lipid bilayers, plasma membrane, or organelle membranes, like pumps or transporters. Apart from these proteins, intracellular Ca^2+^ is regulated by inter-organellar transport and the influx of Ca^2+^ ions from extracellular space [5]. In this review, we provide an overview of key components and the associated major mechanisms of intracellular Ca^2+^ regulation under physiological conditions. It is followed by delineating how these proteins and pathways are manipulated by cancerous cells during tumorigenesis and progression.

Intracellular Ca^2+^ levels are managed through binding to special proteins or sequestration within different cellular compartments. The three main ways by which intracellular Ca^2+^ is buffered are depicted above—soluble or unbound proteins that are found in the non-membranous parts of a cell (cytosol or inside organelles), membrane-bound or intramembranous proteins (generally Ca^2+^ channels (like TRP), ATP-driven pumps (SERCA or PMCA), and ion exchangers (NCX)), and organellar compartments such as endoplasmic reticulum (ER), mitochondria, acidic vesicles (mainly lysosomes and Golgi bodies) or organellar junctions (endoplasmic reticulum-plasma membrane (ER-PM), endoplasmic reticulum-mitochondria, or endoplasmic reticulum-lysosomes) [3,4,5]. The major players regulating inter-organellar Ca^2+^ are mentioned in parenthesis within the cellular organelles section. IP_3_R, inositol-3,4,5-triphosphate; NCX, sodium-Ca^2+^ exchanger; ORAI1 (or CRACM1), Ca^2+^ release activated modulator 1; PMCA, plasma-membrane Ca^2+^ ATPase; SERCA, sarco-endoplasmic reticulum Ca^2+^ ATPase; STIM1, stromal interaction molecule 1; SOCE (store-operated Ca^2+^ entry); TPC1/2, two-pore channel; TRP, transient receptor potential; VDAC, voltage-dependent anion channel.

## 2. Intracellular Ca^2+^ Buffers in Normal Cells

### 2.1. Soluble and Unbound Intracellular Proteins: Calmodulin, Calbindin, and Calretinin

**Figure 2:**
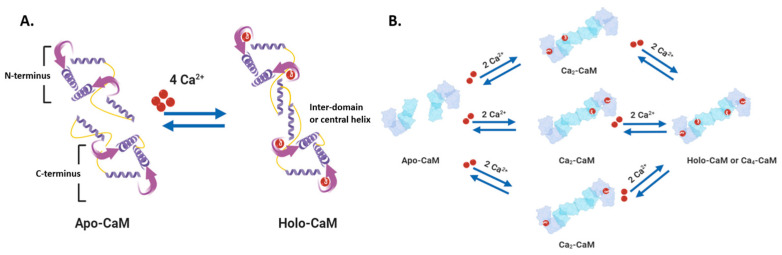
Overview of calmodulin structure and Ca^2+^ binding conformations. (**A**) Calmodulin (CaM) structure comprises four EF-hand structures, two each on N- and C-termini that are connected via an inter-domain helix [6]. CaM exists either as Apo-CaM (no Ca^2+^ bound to EF-hands) or Holo-CaM (Ca_4_-CaM or Ca^2+^ ions bound to all EF-hands); (**B**) Different intermediate calmodulin conformations (Ca_2_-CaM) between Apo-CaM and Holo-CaM with Ca^2+^ ions binding to two EF-hands during each step [8].

The non-membranous proteins inside a cell can act as both Ca^2+^ sensors and buffers [2,3]. Most of these proteins have EF-hand motif(s) that allows Ca^2+^ ions to bind and trigger changes in protein folding, thereby influencing downstream or linked cellular pathways [3,4]. Calmodulin (CaM) is one of the best-studied and ubiquitously expressed Ca^2+^-sensing proteins known to play a key role in intracellular Ca^2+^ homeostasis [3]. As the prototype for intracellular Ca^2+^ sensors, its 148 amino acid structure is comprised of two Ca^2+^-binding sites, each with two EF-hand motifs: N- and C-termini alpha-helices with a Ca^2+^ coordination loop in between providing affinity for Ca^2+^ ion docking and sequestration [6]. The ability of CaM to transmit a change in free intracellular Ca^2+^ levels into modulation of a cellular response comes through its Ca^2+^-dependent structural flexibility. CaM can exist in a Ca^2+^-free closed conformational state (Apo-CaM), a semi-open (Ca2-CaM), or an open state (Holo-CaM or Ca4-CaM) after Ca^2+^-binding [6,7,8] (Figure 2). The latter two conformational states expose hydrophobic residues of this protein, thus allowing it to bind to target or effector molecules and acting as a fast-acting intermediary between change in intracellular Ca^2+^ and cellular processes. Differential Ca^2+^-binding on the two lobes of CaM makes fast buffering of a wide range of free intracellular Ca^2+^ possible for this protein. Analysis of CaM kinetics by Faas et al. has revealed that the N-lobe of CaM acts as the first site for Ca^2+^-binding during a massive increase in intracellular Ca^2+^ levels (>100 mM) in a nanodomain [9], whereas the C-lobe, having a higher affinity for Ca^2+^ than the N-lobe, captures Ca^2+^ in its EF-hand motifs when the Ca^2+^ concentration in the pool of intracellular fluid is 1–10 mM and both the motifs have Ca^2+^-bound to them. The presence of methionine residues in its lobes and plasticity of the central linker in its structure also provides CaM with properties to function as an adaptor protein in intracellular Ca^2+^ signaling [10]. CaM can bind to several targets or effector molecules over a variable distance and in multiple orientations to mediate change in intracellular Ca^2+^ signaling. Some major effector proteins that are regulated by CaM binding and are relevant for Ca^2+^ homeostasis include ORAI, EGFR, PI3K, IQGAP, and connexins [1].

Calbindin D-28k is another Ca^2+^-binding protein with six EF-hand motifs that buffers and transports free cytosolic Ca^2+^ but, unlike calmodulin, does not act as a linker or adaptor protein in shaping intracellular Ca^2+^ signaling [11,12]. Additionally, its expression is limited to a few cell types such as mammalian kidney ductal cells, intestinal absorptive epithelia, and neurons. Calretinin or calbindin D-29k, with 58% homology to calbindin D-28k, acts both as a nonlinear Ca^2+^ buffer and sensor predominantly in the neurons [13,14]. Expressed in the kidney and duodenum epithelial cells, calbindin D-9k or S100G is a monomer comprised of two EF motifs [15]. With no known binding partners, it is only considered a Ca^2+^ buffer. ER (endoplasmic reticulum) molecular chaperones, calreticulin (in the lumen), and calnexin (on the membrane) are also known to be Ca^2+^ buffers [5,16].

**Physiological relevance:** CaM is required for spatial and temporal regulation of [Ca^2+^]i as evident by its role in modulation (activation or inactivation) of Ca^2+^ pumps (such as PMCA and SERCA) and Ca^2+^ channels (such as CaV1.3, TRPV5 and 6, ORAI) [17,18,19]. CaM also acts via serine/threonine kinases known as Calmodulin-activated Kinases (CaMKs) to influence cellular processes like proliferation (for example, centrosome duplication at G1/S or anaphase to metaphase transition via CaMKII) [20]. Calbindin D-28k acts as a Ca^2+^ buffer proximal to Ca^2+^ channels like TRPV5 and maintains a steep gradient for ion entry [21].

### 2.2. Intramembranous Molecular Buffers: SERCA, PMCA, NCX, and TRP

**Figure 3:**
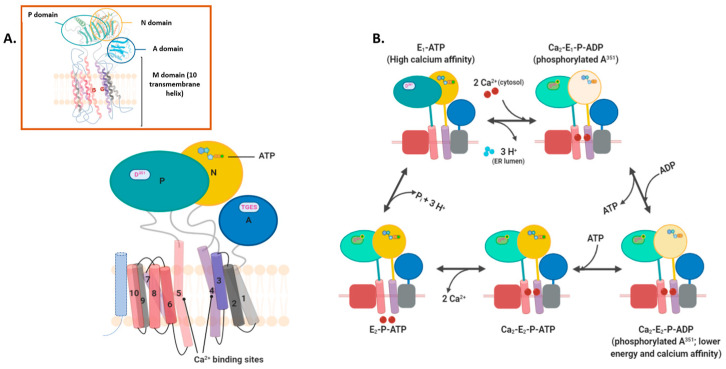
Sarcoplasmic endoplasmic Ca^2+^-ATPase structure and mechanism of Ca^2+^ ion transport. (**A**) SERCA pump structure is comprised of Actuator or A domain (for dephosphorylation), transmembrane or M domain (10 helices and two Ca^2+^ ion binding sites between helices 4 and 5), Nucleotide or N domain (source of phosphorylation), and Phosphorylation or P domain (consists of the site of phosphorylation; aspartate 351 residue) [26]. Picture in the inset is a simplified representation of SERCA crystal structure; (**B**) Numerous steps are involved in the transport of Ca^2+^ via SERCA across the endoplasmic reticulum membrane [28,29,30,31]. As per the above model, the pump cycle begins with SERCA having a high affinity to Ca^2+^ in its physiologically predominant E1-ATP state. After cytosolic Ca^2+^ ions bind to SERCA, the pump transitions into Ca2-E1-P-ADP state utilizing the energy from the transfer of ATP γ-phosphate in the N domain to D351 amino acid residue in P domain. Transport of Ca^2+^ ions to the ER lumen parallels the shift of pump from its high-energy intermediate conformation to the low energy E2-ATP state (with less affinity to Ca^2+^; not shown) triggered by replacement of ADP with ATP. Ca^2+^ ions are then exchanged in the lumen with 2–3 protons compensating for the loss of positive charge. Followed by dephosphorylation at D351. In a Ca^2+^-free resting stage, the γ-phosphate of ATP in N-domain is masked from phosphorylation site (P domain Asp^351^) as well as the dephosphorylation site (A-domain TGES sequence) [25,32]. When two cytosolic Ca^2+^ ions bind to SERCA pump in its higher Ca^2+^ affinity state (known as E_1_-ATP), it results in rotational changes within the transmembrane and cytoplasmic domains, thus bringing N-domain ATP closer to Asp^351^ for transfer of γ-phosphate. From this high-energy intermediate state (Ca_2_-E_1_-P-ADP) the pump prepares to transition into a lower energy state, Ca_2_-E_2_-P-ADP by completely transferring the γ-phosphate to P-domain and coordinating Mg^2+^ ions to all the phosphate groups present. In a two-step process again, first ADP is exchanged for ATP in the ER causing appropriate conformational changes to create a channel and exposing the Ca^2+^-binding residues in its lumen (E_2_P-ATP). Ca^2+^ ions are then released in exchange for protons and the pump restores its resting state. ADP, adenosine diphosphate; ATP, adenosine triphosphate; SERCA, sarcoendoplasmic reticulum Ca^2+^ ATPase.

Intra-membrane Ca^2+^ buffers primarily translocate free Ca^2+^ between domains and organelles. These mainly comprise ion exchangers, channels, and ATP-driven pumps [22]. SERCA or Sarcoendoplasmic Reticulum Ca^2+^ ATPase is an ATP-dependent ion pump known to significantly maintain free cytosolic Ca^2+^ concentration via actively pumping the ion into the endoplasmic reticulum (or sarcoplasmic reticulum in muscle cell). Among the eleven of these P-type ATPase pump isoforms (and variants) recognized so far, SERCA1a and SERCA1b are mainly expressed in adult and neonatal skeletal muscle cells, respectively. SERCA2a is found in cardiomyocytes, while SERCA2b and 2c are expressed ubiquitously. SERCA3 (all the six splice variants) are largely co-expressed with SERCA2b in hematopoietic, endothelial, and epithelial cells. With 85 percent and 75 percent overlap of SERCA2 and SERCA3 primary sequences to that of SERCA1, these isoforms exhibit differential affinity for Ca^2+^; SERCA3 demonstrates a fivefold lower propensity to bind Ca^2+^ than other isoforms [23,24,25]. Regardless, the isoforms share a general structure that includes 10-pass transmembrane helices and three cytoplasmic domain lobes (Figure 3A) [26,27]. Two closely spaced Ca^2+^-binding sites are present on the cytoplasmic side of transmembrane domains. These sites act cooperatively with each other such that the binding of Ca^2+^ ions to site I increases the binding affinity for site II [27]. Once the two Ca^2+^-sites are occupied, the cytoplasmic lobes—the nucleotide-binding (N)-domain followed by phosphorylation (P), and actuator (A)-domains—undergo conformational shifts and translocate Ca^2+^ ions [25,26] (Figure 3B).

Although phospholamban (PLN) has been shown to be a stronger inhibitor of the pump, some studies indicate sarcolipin (SLN) to inhibit SERCA at high Ca^2+^ concentrations [24,28]. Both PLN and SLN are type I transmembrane micropeptides that bind as dephosphorylated monomers (active form) to a groove surrounded by TM2, TM4, TM8, and TM9 of SERCA. SERCA2b being the more widely expressed isoform in non-muscle cells is modulated by other means than PLN and SLN. The inhibitors include another-regulin (ALN; a ubiquitously expressed inhibitor with PLN/SLN key SERCA2b-interacting amino acids), an additional transmembrane helix (TM11), and a cytoplasmic end luminal extension of SERC2b [28] (Table 1).

**Figure 4:**
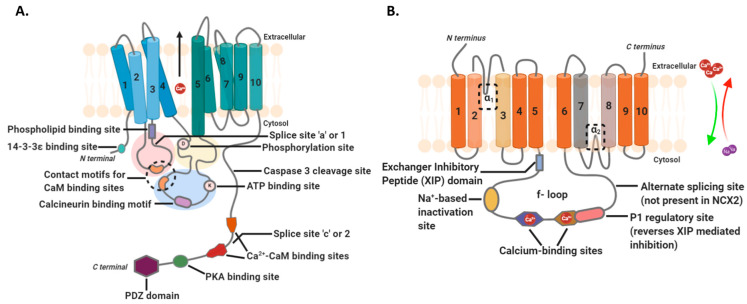
Structures of intracellular Ca^2+^ efflux machinery. (**A**) Plasma membrane Ca^2+^-ATPase (PMCA) is comprised of cytosolic N- and C-termini, two Ca^2+^-binding sites (akin to SERCA; not shown here), and 10 transmembrane (TM) segments. Between the N- and C-tails lie three loop structures named like the domains in SERCA—A domain (red circle between TM 2–3), P domain (blue circle between TM 4–5), and N domain (yellow circle between TM 4–5) [34,36,37,38,39,40]. Various binding motifs and regulatory or interaction sites exist on each terminus and cytosolic domain with variations observed in their presence between PMCA isoforms and splice variants. Ca^2+^-bound calmodulin or Ca^2+^-CaM is a major regulator for all PMCA family members. The proximity of Ca^2+^-CaM binding sites on the C-tail to their corresponding motifs in A- and N-domains determine the open or closed states of the pump; (**B**) Sodium–Ca^2+^ exchanger (NCX) also has a ten transmembrane topology with N- and C-tails facing the extracellular space [42,43,44,45,49]. The cytosolic side consists of an f-loop (between TM 5–6) that has two Ca^2+^ ion binding sites, an alternate splicing site, a regulatory site, a sodium ion-dependent inactivation site, and an inhibitory domain. The structure also has two conserved alpha-helical repeats (α1 and α2) that influence ion binding and transport. ATP, adenosine triphosphate; CaM, calmodulin; NCX2, sodium-Ca^2+^ exchanger 2; PKA, protein kinase A; PDZ, a structural domain; XIP, exchanger inhibitory peptide.

P-type Ca^2+^-ATPases also exist within the plasma membrane and maintain cytosolic Ca^2+^ levels by transferring them into the extracellular space. The Plasma Membrane Ca^2+^ ATPases (PMCAs) were earlier known only as housekeeping proteins required for intracellular Ca^2+^ homeostasis, but some isoforms and splice variants are now known to have a more active role [33,34]. PMCAs transport one Ca^2+^ ion per ATP molecule which differs from two Ca^2+^ ions per ATP molecule stoichiometry of SERCA [35]. Four PMCA isoforms are known in mammals, each one with many splice variants [34]. PMCA1 (especially PMCA1b) has a ubiquitous expression with its presence being essential even during embryonic development. PMCA2 and PMCA3 are expressed more selectively in tissues such as the brain, pancreatic β cells, inner ear cells, mammary glands, and the heart. The pattern of PMCA4 tissue expression of overlaps with that of PMCA1, however, its absence does not cause embryonic lethality. The isoforms and variants also differ in the activation/inactivation rates which makes it possible for PMCAs to manage intracellular Ca^2+^ in the form of fast-acting spikes or slowly released puffs [34,36]. The general structure of such Ca^2+^ transporters comprises 10 transmembrane segments with large cytosolic loops TM 1–2 and TM 3–4, a cytosolic N- and C-termini tails [34,36,37] (Figure 4A). The first cytosolic loop consists of splice site A (regulates membrane-targeting) and phospholipid-binding sites, the second loop has an aspartyl-containing phosphorylation site, and the C-terminus is strewn with the calmodulin-binding site, splice site C and PDZ domain. The activity of all PMCA isoforms (and variants) is heavily regulated. Short-term regulation of catalytic activity of most of the “b” or splice site 2 variants is mainly calmodulin-dependent [37,38,39,40]. The binding of CaM reverses auto-inhibition of the pump due to conformational shifts which displace C-tail from cytosolic loops. Other means of autoinhibition reversal include phosphorylation of C-tail (Ser/Thr residues) by protein kinase A or C, proteolytic cleavage of C-tail, or dimerization via C-terminus. Change in the localization via interaction with PDZ proteins like MAGUK (membrane-associated guanylate kinase) at the C-tail or transcriptional and translational modulation influences the long-term activity of PMCAs.

PMCAs also partner with sodium-Ca^2+^ exchanger (NCX) in some cells to remove Ca^2+^ from the cytosol [41]. PMCA has high Ca^2+^ ion affinity and low capacity when compared to NCX that has low Ca^2+^ ion affinity but a high capacity for ion efflux. This means that PMCA maintains basal cytosolic levels or small bursts of Ca^2+^ ion entry while NCX is responsible for regulating large but transient increase in intracellular Ca^2+^. NCX or SLC8 belongs to a superfamily of the Ca^2+^ ion/cation antiporter gene family. Within the SLC8 family, NCX1, 2, and 3 are the identified functional members encoded by separate genes in mammals [42]. NCX1 is expressed ubiquitously, NCX2 is found in the brain and skeletal muscles, and NCX3 in neurons. Topological analysis of this family based on NCX1 predicts a structure with ten transmembrane alpha-helices. The first five helices form the N-terminus which is separated by a cytosolic loop from the remaining helices forming the C-terminus. The cytosolic loop (500 a.a.) is a regulatory site—a beta repeat region with two Ca^2+^ ion binding regions, CBD1 and CBD2. CBD1 is the primary site for detecting small changes in cytosolic Ca^2+^ ion concentration resulting in greater structural changes that activate the exchanger. CBD2 only responds to moderate change in cytosolic Ca^2+^ ion levels [42,43,44,45] (Figure 4B).

**Physiological relevance:** Blocking the function of SERCA isoforms can lead to disproportionate Ca^2+^ levels in the cell cytosol, thus activating apoptosis signals in select cell types. For instance, reduced SERCA2b activity in hepatocytes results in ER stress followed by cell death due to accumulation of excessive cytosolic Ca^2+^ [46]. PMCA can help maintain the local intracellular Ca^2+^ ion [Ca^2+^]i gradient required for cellular motility. Migrating endothelial cells have higher expression of PMCA at their leading edges to maintain low basal [Ca^2+^]i levels thereby, preventing continued activation of Myosin Light Chain Kinase (MLCK) and extended contraction of the cell membrane at the migration frontier [47]. On the other hand, NCX1 inhibition and thus impairment of [Ca^2+^]i extrusion allows the proliferation of pancreatic beta cells [48].

**Figure 5:**
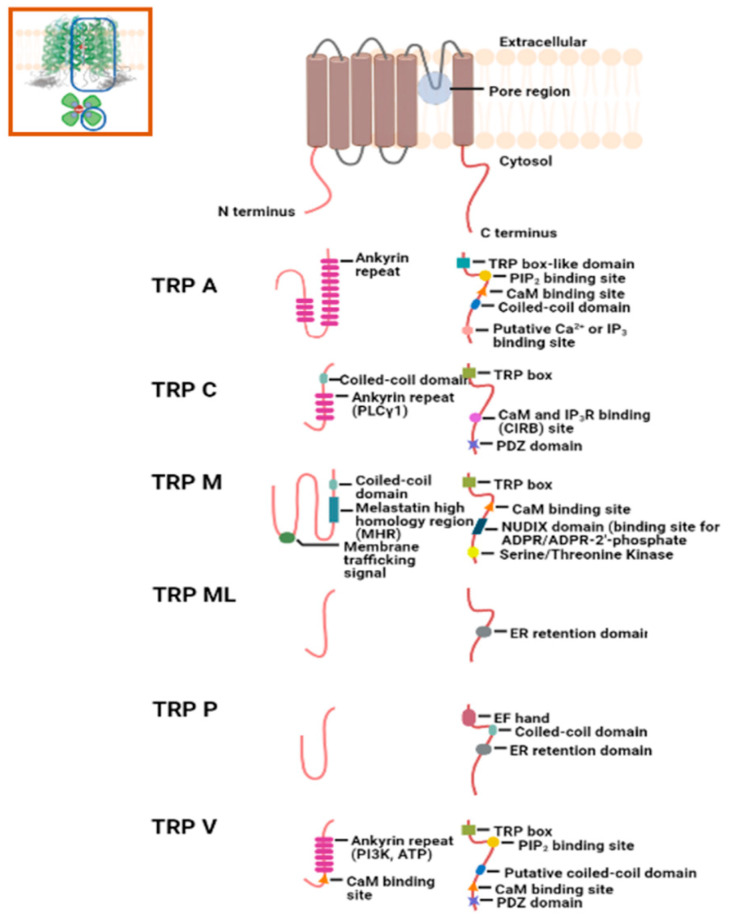
Structure of TRP channel isoforms. The general topology of a TRP channel includes a homo- or hetero-tetrameric formation (see inset). The basic structure of each subunit consists of cytosolic N- and C-termini, and six transmembrane domains with the pore region between TM 5–6. A variable number of ankyrin repeats on the N-terminus are found in TRP A, TRP C, and TRP V. No specific interaction site or functional domain has been identified so far on the N-terminus of TRP ML and TRP P. The most common domains found on the C-terminus across TRP isoforms are TRP box, coiled-coiled domain, and calmodulin (CaM) binding site [59,60,61,62]. ADPR, adenosine diphosphate ribose; CIRB, calmodulin and IP_3_R binding site; NUDIX, nucleoside diphosphate-linked moiety X; PI3K, phosphoinositide 3-kinase; PIP_2_, Phosphatidylinositol 4,5-bisphosphate; PLCγ, phospholipase C gamma.

Initially ascribed to Ca^2+^ homeostasis in sensory neurons, Transient Receptor Potential (TRP) channels have lately been appreciated for a similar function in epithelial and immune cells [50]. Mammalian TRP channel superfamily is composed of 28 family members belonging to six subfamilies—TRPC (Canonical), TRPA (Ankyrin), TRPM (Melastatin), TRPV (Vanilloid), TRPP (Polycystin), and TRPML (Mucopilin)—that differ in their sensitivity to various sensory stimulations and affinity for cations (including Ca^2+^ ions) sequestration [51]. Commonly, TRP family members share a structure with six transmembrane domains, intracellular N- and C-termini, and a pore-forming TM 5–6 loop [52] Figure 5. Cation selectivity of a TRP channel is determined via an extracellular portion of the pore-forming TM 5–6 helices [51,53]. To have a functional channel for Ca^2+^ transport, TRP members form homo- or hetero-tetramers within and across the subfamily members [54]. The N-terminus of each tetramer subunit of a TRP channel along with corresponding transmembrane helices are associated with channel assembly and pore regulation [55]. The N-terminus within and across each TRP subfamily varies in the presence and number of ankyrin repeats; having such repeats in general at the amino end provides a site for protein-protein interaction or ligand binding [51,56,57]. The intracellular C-terminus of each subunit is a site for protein interaction and post-translational modification. It also brings structural and functional diversity between subfamilies [52,53,58]. For example, TRPC, TRPV, and TRPM subfamilies share a C-terminus TRP box motif—a short cytosolic hydrophobic stretch at the end of the transmembrane domain putatively holding the channel in a closed conformation [53,59]. Moreover, the C-tail of these channels can have PDZ protein binding domains (TRPV and C), sites for interaction with G-proteins (Gq/11)/calmodulin/PLCβ, ADP-ribose binding (NUDIX; TRPM2), or PLC-interacting kinase (PLIK; TRPM6 and 7) domains [59] (Table 2).

**Figure 6:**
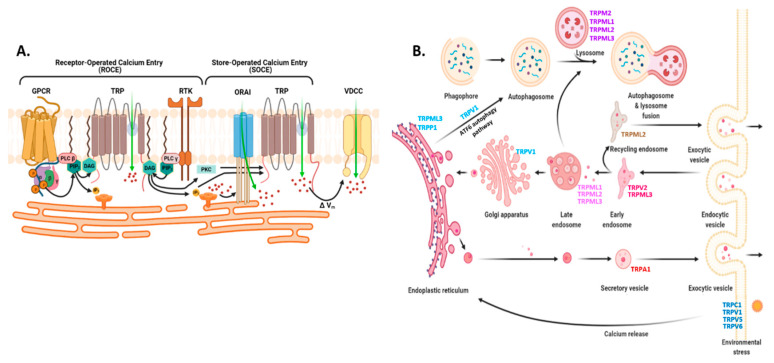
Many roles of TRP channels in intracellular Ca^2+^ homeostasis. (**A**) TRP channels drive Ca^2+^ influx either through Receptor-Operated Ca^2+^ Entry or Store-Operated Ca^2+^ Entry. ROCE requires activated GPCRs or Receptor-tyrosine kinases to initiate phospholipase C beta (PLC β) or PLC γ mediated hydrolysis of phosphatidylinositol 4,5-bisphosphate (PIP_2_) into diacylglycerol (DAG) and inositol 1,4,5-triphosphate (yellow circles) after which DAG binds to a TRP channel to stimulate the inflow of Ca^2+^ ions. IP_3_ generated via PIP_2_ hydrolysis triggers a reduction in Ca^2+^ ion levels in the endoplasmic reticulum by simultaneously promoting the release of the cations through IP_3_ receptors (IP_3_Rs) [54,63,69,71,72,76,77,78]. This results in the formation of SOC complex (ORAI-STIM or ORAI-TRP-STIM) for transporting Ca^2+^ ions from extracellular space into the cytosol. Some TRP isoforms can also be activated by direct binding of protein kinase C that triggers Ca^2+^ influx (or sodium influx) followed by membrane depolarization leading to more Ca^2+^ influx via the opening of voltage-dependent Ca^2+^ channels [54]. Other TRP isoforms (especially expressed in muscle cells) can directly induce Ca^2+^ release from IP_3_Rs on the ER membrane (Ca^2+^-induced Ca^2+^ release or CICR; not shown); (**B**) TRP channels are expressed on intracellular membranes as well where they play an active role in the regulation of Ca^2+^ ion concentration during processes such as autophagy, endocytosis, and exocytosis (58,61,75). DAG, Diacylglycerol; GPCRs, G-protein coupled receptors; IP_3_, inositol 1,4,5-triphosphate; ORAI (Ca^2+^ release activated Ca^2+^ modulator; RTKs, Receptor-tyrosine kinases; ROCE, Receptor-Operated Ca^2+^ Entry; SOC, store-operated Ca^2+^; SOCE, Store-Operated Ca^2+^ Entry; STIM, stromal interaction molecule; PIP_2_, phosphatidylinositol 4,5-bisphosphate; PKC, protein kinase C; TRP, transient receptor potential; VDCCs, voltage-dependent Ca^2+^ channels.

**Physiological relevance:** On a high level, TRP channels act as activators, integrators, as well as downstream effectors of Ca^2+^ signaling at the plasma membrane and in intracellular compartments [54,55,69] (Figure 6). Almost all TRP channels permeable to Ca^2+^ ions (other than TRPM4 and 5), can directly activate intracellular Ca^2+^ signaling [51]. Taking TRPC as an example, many members of this subfamily are activated by DAG (Diacylglycerol) which is produced by PLC β- or γ-mediated cleavage of phosphatidylinositol 4,5-bisphosphate (PIP_2_) after the ligand binding at GPCRs or RTKs [59]. Being a family of non-selective cation channels reacting to numerous stimuli, TRP channels can also indirectly influence the activation of receptor-operated bulk entry of Ca^2+^ from the extracellular space [58]. TRPP1/2, TRPA1, TRPM8, and TRPV1-4 are all expressed on the ER membrane [54,70]. At this site, PLC-independent activation of the TRP channels (such as TRPV1) is suggested to induce ER Ca^2+^ release via inositol triphosphate receptor (IP3R) which further triggers bulk entry of extracellular Ca^2+^ into the cell (discussed further in a later section) [71,72,73,74]. On the flip side, cytosolic Ca^2+^ regulates the activity of TRP channels in response to physiological stimuli. This regulatory effect is usually through CaM binding (inhibition of TRPV5, TRPV6, and sensitization of TRPV3) and indirectly through CaM-binding kinase II (CaMKII) [69]. Among the extra-neuronal TRP channels, TRPA1, TRPC1, TRPM8, TRPV1, and TRPV4 have been recognized for their role in epithelial and immune cell Ca^2+^ homeostasis [50,75]. By regulating intracellular Ca^2+^, some of these channels like TRPV1 and TRPM8 which are expressed in human bronchial and lung epithelium, respectively, aid the release of chemoattractants and promote immune cell–epithelial cell interaction.

### 2.3. Cellular Organelles

#### 2.3.1. Endoplasmic Reticulum: STIM, ORAI, IP_3_Rs, and TRPC1 in SOCE and SOCIC Ca^2+^ Entry Models

The ER serves as the largest and most dynamic organelle reservoir for intracellular Ca^2+^ and is therefore central to an array of cell signaling processes for protein synthesis, folding, and post-translational modifications [79,80,81,82]. In contrast to the cytosol, ER Ca^2+^ ion levels can range from 100 uM to 1 mM based on the cell type [72,80]. ER and other intracellular organelles buffer excessive cytosolic Ca^2+^ by both housing Ca^2+^-binding proteins (example: calreticulin in ER) and via active transport (example: SERCA pumps in ER) [5,79,80]. Depletion of Ca^2+^ from the ER lumen actuates an indirect mode of Ca^2+^ entry into the organelle which is termed Store-Operated Ca^2+^ Entry (SOCE) or Ca^2+^ Release Activated Ca^2+^ (CRAC) entry; it is activated when plasma membrane receptors like PLC-coupled GPCRs (but not voltage-gated channels) trigger Ca^2+^ ion release from the organelle [83,84]. Exhaustion of the intraluminal ER Ca^2+^ ion store following such prolonged release is then sensed by STIM (Stromal Interaction Molecule) tethered to the ER membrane and subsequently relayed to the CRAC channels on the plasma membrane.

**Physiological relevance:** SOCE and its key players participate in multiple normal cellular processes that go awry during cancer progression. For example, siRNA mediated downregulation of STIM1 and ORAI1 in keratinocytes impairs cellular differentiation [85], whereas SOCE is found to be inactivated during mitosis [86]. By contrast, stimulation of IP_3_Rs (and RyRs) promotes cell cycle progression of stem cells, pancreatic beta cells, renal cells, and more.

**Figure 7:**
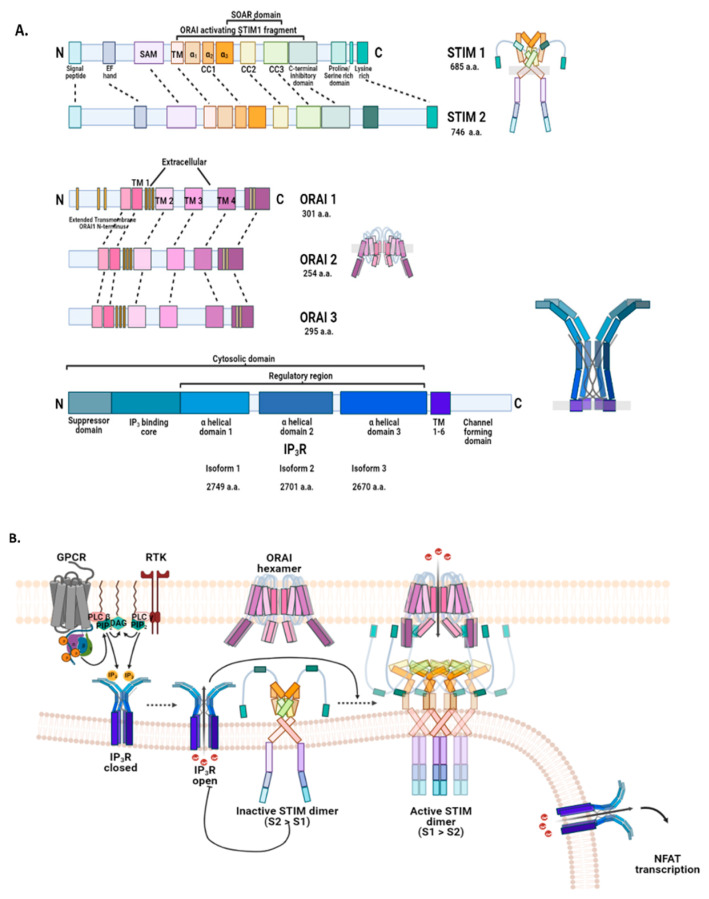
Major molecules in store-operated Ca^2+^ entry. (**A**) STIM, ORAI, and IP3Rs are central to the influx of Ca^2+^ ions from extracellular space into cytosol re-quired for replenishing the depleted intracellular stores. In humans, STIM has two isoforms. Both, STIM1 and STIM2 have a type I transmembrane structure comprised of a signal peptide at the N-terminus for localization to the ER, an EF-hand motif, and a sterile alpha motif for binding of luminal Ca^2+^ ions. SAM is also a site for protein–protein interactions), and a short transmembrane segment followed by three coiled-coiled domains (CC1, CC2, and CC3, that form ORAI-activating STIM fragment or OASF). CC2-CC3 region (or SOAR) is adequate as well for STIM–ORAI interactions [106,107,108,109]. The ORAI interacting sites on the domain are hidden in inactive STIM dimers. A Ca^2+^-dependent inhibitory domain, a proline/serine-rich region (polybasic; PBS), and a lysine-rich tail segment are found at the C-terminus. ORAI channels have three isoforms in humans. All the ORAI isoforms have an N-terminal ETON segment (cytosolic), four transmembrane domains, and a cytosolic C-terminus. The structure between TM 1–2 and TM 3–4 is exposed to the extracellular space. The yellow vertical bars depict regions of amino acid residues (positive, negative, hydrophobic, or special) that influence ORAI channel pore selectivity. ORAI channel exists as a hexamer that is formed by oligomerization of 3 dimer subunits. Inositol 1,4,5-triphosphate receptor subunits have most of their structure (5 domains) exposed to the cytosol. Each IP3R is formed of four subunits [110,111,112,113]. The transmembrane segments and the C-terminus are involved in pore-forming, gate-keeping, and tetramerization; (**B**) Subsequent to the activation of IP3Rs on the ER membrane by ligand IP3 and cytosolic Ca^2+^, STIM dimers are activated once the luminal Ca^2+^ concentration drops below basal levels [114,115,116,117,118,119]. The coiled-coiled domains experience a shift in their conformation, thus exposing the SOAR/OASF region for interaction with ORAI channels on the plasma membrane [120]. STIM-ORAI functional coupling for SOCE pore formation requires 3 STIM dimers interacting with one ORAI hexamer [87]. STIM dimer composition and function vary under different cellular states. Under resting or physiologically inactive state (or with low stimuli), STIM dimers are largely composed of STIM2 (S2) which has an inhibitory effect on IP3R activation. As the activation stimuli become stronger and intracellular store gets depleted of Ca^2+^, STIM dimers composed mostly of STIM1 takeover to participate in SOCE. IP3Rs are also expressed on parts of ER membrane away from the ER-PM microdomain involved in SOCE. These receptors provide intracellular Ca^2+^ ions for downstream Ca^2+^ signaling including NFAT-mediated transcription. Red semi-circle in the ER represents high luminal Ca^2+^ levels, pink semi-circle is for moderately low Ca^2+^ ion concentration, and pale semi-circle indicates extremely low Ca^2+^ concentration. CC, coiled-coil; NFAT, nuclear factor of activated T-cells; SAM, sterile alpha motif; SOAR, STIM1 Orai1-activating region; TM, transmembrane.

STIM, a type I transmembrane protein, was originally identified during a search for transmembrane and secretory proteins in stromal and pre-B lymphocytes [84,87]. The role of STIM in [Ca^2+^]_i_ signaling was later confirmed by independent work of two groups using high-throughput RNAi screens to identify inhibitors of thapsigargin evoked CRAC current (ICRAC) [88,89,90]. Both the isoforms, STIM1 and STIM2 have a luminal N-terminus and a cytoplasmic C-terminus [91]. Starting at the N-terminus, the basic STIM structure is comprised of EF-hand motifs (canonical and non-canonical) and a sterile α-motif (SAM), all of which help in intraluminal Ca^2+^ ion sequestration [83,88]; Figure 7A. The C-terminus has various coiled-coiled domains (CC1-3) that include CRAC activating domain (CAD; also known as STIM-ORAI activating region (SOAR)), and a polybasic site (PBS). The cytoplasm-facing C-terminus of STIM is essential for localization to ER-PM junctions and for interaction with CRAC channels. Even with greater than 74 percent sequence similarity, the STIM isoforms perform differently as ER Ca^2+^ sensors—the Ca^2+^ affinity of EF-SAM domains being greater for STIM1 (200 mM; 400 mM for STIM2) while the time to form oligomers for plasma membrane CRAC channel association is nearly 70 times longer for STIM2 [84,91,92,93]. This makes STIM2 more suitable for the regulation of basal ER Ca^2+^, although only a small contribution to SOCE was recorded in some studies [54,76]. In fact, STIM2 has been shown to inhibit STIM1-mediated SOCE [94]. With the expression of STIM1 on both the plasma membrane and the ER membrane, the difference between the cellular localization of these isoforms also highlights their functional disparity [90].

A year after STIM molecules were recognized as ER Ca^2+^ ion sensors, ORAI1 was confirmed as Ca^2+^ Release-Activated Ca^2+^ Modulator 1 (CRACM1) or simply, the CRAC channel [84,95]. Genome-wide RNAi screening, linkage analysis, and positional cloning isolated and identified ORAI1 as the mutated gene responsible for severe combined immunodeficiency in humans (with low ICRAC in T lymphocytes). Three isoforms of ORAI proteins exist in mammals—ORAI1, ORAI2, and ORAI3 [95,96]. Topologically, an ORAI channel-forming monomer has four transmembrane domains forming two intracellular (I-II, III-IV) and one extracellular peptide loop (II-III) [97,98] (Figure 7A). Both N- and C-termini are cytoplasmic with each having a STIM1-binding site or CRAC activation domain (CAD; 73–91 a.a. on amino side and 268–291 a.a. on the carboxyl side of ORAI1). A Ca^2+^-binding site is present at E106 that resides in the conserved TM1 segment. The three isoforms have high sequence homology in the transmembrane domains (92%) but only 62 percent overall due to some differences in the C-terminus (coiled-coiled domain) and III-IV loop. This results in variations between the isoforms in terms of activation time, Ca^2+^ ion-dependent inactivation kinetics, activation, or inhibition by 2-aminoethoxydiphenyl borate, affinity for STIM1, redox sensitivity, and activation by STIM2 [96,99,100].

The mechanistic model of SOCE has evolved over the last two decades. Going by the widely accepted “interaction model” or “diffusion trap model”, STIM1 exists as a dimer in resting (inactive) state where the Ca^2+^-bound canonical EF-hand, the non-canonical EF-hand, and SAM domain on each monomer together impart structural stability [95,99,100] (Figure 7B). It is complemented by the conformation of the cytosolic segment that keeps the ORAI-binding CAD domain, and the plasma membrane interacting polybasic C-tail hidden away. Depletion of unbound ER Ca^2+^ then triggers the freely diffusing STIM1 dimers on the ER surface to lose Ca^2+^ ions from cEF; this causes a conformational change on the C-termini exposing STIM CAD domains and extending the polybasic tails to bind ORAI hexamers and PIP2, respectively, at the ER-PM junction. Some studies have recognized STIM1 expressed on PM along with ORAI1 as key players for a store-independent, arachidonic acid-regulated Ca^2+^ current in association with ORAI3 [101,102]. Nonetheless, the function of plasma membrane STIM1 is debatable to date.

The release of intraluminal ER Ca^2+^ that evokes an influx of extracellular Ca^2+^ via ORAI1-STIM1complex at the ER-PM puncta is best known to be mediated by activated IP3R [103,104]. Certain types of ligand-stimulated GPCRs and RTKs generate IP3 and DAG as secondary effectors; the IP3 molecules then bind to its receptors expressed on the ER surface. Sampieri and Vaca et al. discovered IP3Rs to be spatially and functionally associated with STIM1 localized at the puncta [105]. In their study, activated IP3Rs immediately localized to STIM1 to form a complex that allowed the cEF domain of the latter to effectively sense nearby intraluminal Ca^2+^ depletion. Furthermore, overexpression of IP3Rs was shown to enhance the SOCE Ca^2+^ influx. Contrastingly, IP3Rs remain inactive during ER Ca^2+^ leak due to SERCA pump inhibition. Consequently, Ca^2+^ ion depletion around STIM1 cEF is slow and ineffectivewhich leads to smaller ORAI1 currents.

Mikoshiba et al. were the first to identify and characterize IP3Rs in Purkinje cells of cerebellar mutated mice as binding sites for the second messenger, IP3 [121]. Three members of this receptor have been identified in mammals all of which share a 2700 amino acid structure composed of six transmembrane segments and five distinct domains—IP3-binding suppressor/coupling domain at N-terminus, IP3-binding domain, internal modulatory/coupling domain, Ca^2+^ pore-forming transmembrane domain, gate-keeper domain at the C-terminal [122] (Figure 7A). Four IP3R subunits, each with the five domains, come together to form a functional IP3R in a homomeric or heteromeric fashion. Other than the pore-forming domain, the majority of the IP3R structure is extracellular (Figure 7A). The affinity of the IP3-binding domain for IP3 is similar between the isoforms. However, the binding affinity is modified by the N-terminal coupling domain [123]. It has been now revealed that the 225 a.a. segment in the N-terminus of IP3R1 decreases the binding of IP3 to the receptor-binding site by directly interacting with it. Different studies agree that IP3R2 is the most sensitive and IP3R3 is the least sensitive for IP3 docking [122]. The N-terminal domain also allows for receptor regulation via proteins like HOMER, CaM, Ca^2+^ Binding Protein 1 (CaBP1), Ankyrin, and IRBIT (IP3R binding protein released with inositol 1,4,5-trisphosphate) [124,125,126,127,128,129]. IP3 and Ca^2+^ ions are the main regulators of IP3R channel activity [123]. Each subunit of the functional IP3R tetramer has a binding site for IP3 and the predominant observation is that channel opening requires occupancy of more than one but not all four IP3-binding sites. N-terminal residues 1–223 and internal modulatory domain residues 651–1130 have been shown to be essential for coupling IP3 binding to channel opening [130]. Studies have found IP3 docking to also be necessary for IP3 receptor cluster formation in the ER membrane for localized Ca^2+^ release and SOCE activation [131,132]. Regulation by cytosolic Ca^2+^ ions is biphasic; a minor increase in its concentration accelerates channel activity in response to IP3 while higher levels inhibit channel opening [72,122,133]. Purportedly, intraluminal Ca^2+^ influences IP3 binding to the receptor as well with high levels sensitizing the ligand–receptor interaction and vice versa [133]. IP3R-mediated Ca^2+^ release has another positive regulator, OAG (1-oleoyl-2-acyl-sn-glycerol) that indirectly amplifies the channel activity by increasing IP3 production through the PLC pathway [134].

Physiologically, intracellular Ca^2+^ release by IP3R and store refilling by SOCE occur less in phasic format and overlap to quite an extent [88]. This means that in between the IP3-dependent Ca^2+^ ion leaks, closure of IP3Rs, and initiation of Ca^2+^ influx by SOCE, there is a time window when some IP3Rs are active and releasing Ca^2+^ while ion influx by STIM-ORAI complex and SERCA pump-mediated ER store repletion are occurring. The active IP3Rs in that duration are, however, not clustered the ER-PM junction and thereby contribute to differential activation of SOCE downstream effectors situated near puncta but away from the Ca^2+^ influx nanodomain [88,98]. Apart from IP3R, the translocon, which is a complex of proteins that help newly formed polypeptides having signaling sequence to be transported from cytosol to ER, acts as another Ca^2+^-leak channel [135]. When bound to a ribosome, a translocon complex forms a leak pore of 4–5 nm in size that is permeable to intraluminal Ca^2+^ during the resting state of ER.

Newer studies have provided evidence for more proteins besides STIM, ORAI, and IP3R to be participants in store-operated Ca^2+^ entry. Luis Vaca was the first to describe a protein complex termed Store-Operated Ca^2+^ Influx Complex or SOCIC that involves many key proteins such as TRPC1, SERCA, and microtubule end-tracking protein, EB1 [136]. The quintessential CRAC channels required for SOCE have a voltage-independent, highly Ca^2+^ ion-selective, inwardly rectifying (reversal potential near 40 mV), low amplitude (6 fA) current at physiological negative membrane potential which is blocked by low concentration of lanthanides and a high dose of 2-APB (2-aminoethoxydiphenyl borate) [137]. On the other hand, the delayed global Ca^2+^ influx mediated by SOCIC complex that simultaneously utilizes TRPC as its ion channel generates a current amplitude up to 1–2 pA [84]. TRPCs were originally speculated to be CRAC channels [138]. Typically, electrophysiological properties of TRPC homomers include an inwardly rectifying (reversal potential about 15 mV) Ca^2+^ ion-selective current that is activated by store depletion (via thapsigargin or IP3 inclusion in micropipette during whole-cell patch clamp) and inhibited by lanthanides as well as 2APB [139]. Experimental evidence has also demonstrated a decline in SOCE response corresponding to downregulation or no expression of endogenous TRPCs in vitro and in vivo, respectively. Nonetheless, certain dissimilarities in their current properties with that of ICRAC made the role of these channels in SOCE debatable. Presently, TRP channels are known as the key components of receptor-operated Ca^2+^ entry (ROC) where these channels open for Ca^2+^ entry in response to increased DAG levels from PIP2 hydrolysis upon Gq-coupled receptor activation [139]. However, with the proposed SOCIC model and some recent studies, the contribution of TRPC channels to ISOC currents is beginning to unravel [136,139].

The SOCIC model is based on the findings that TRPC-mediated Ca^2+^ influx is dependent on several interactors [138]. STIM1 is a major interactor and regulator of TRPC expressed within the ER-PM junctions. Vesicular TRPC1 is positioned near ER–PM junctions in unstimulated cells with the help of Rab4-dependent fast-recycling endosomes [140]. Store depletion and subsequent Ca^2+^ entry by ORAI1-STIM1 complexes translocate vesicular TRPC1s into the proximity of ORAI1-STIM1 clusters present on the cell surface. At this point, critical interaction between TRPC1s and caveolin-1 allows insertion of these channel proteins into cholesterol-rich lipid rafts in the junctions, thereby placing them proximal to ORAI1 for subsequent activation by Ca^2+^ influx. To be activated and participating as a store-operated Ca^2+^ channel, TRPC1 in the lipid rafts dissociates from caveolin-1 before engaging STIM1. STIM1 gates TRPC1 through the interaction of lysine residues at 684–685 a.a. in its polybasic tail with the aspartate residues at 639–640 a.a in the tetrameric channel protein. Other regulatory interactions between these proteins involve STIM1 ERM and SOAR domains, and TRPC1 coiled-coiled domains. Following a sustained and global Ca^2+^ release inside the cells, TRPC1 dissociates from STIM1 to re-associate with caveolin-1 for internalization into Rab5-linked early endosomes (via Arf6-dependent pathway). Due to the experimental proof of direct interaction between ORAI1-TRPC1-STIM1 shown by coimmunoprecipitation, Luis Vaca et al. considered TRPC1 to be the pore-forming component and the surrounding ORAI1 to be the regulator in their proposed SOCIC model [136]. Lately, studies have diverged from this model with mounting evidence supporting the formation of distinct but interacting ORA1-STIM1 and TRPC1-STIM1 Ca^2+^ ion conducting pores within the lipid rafts [139,141]. Using the new model, ORAI1-STIM1 based Ca^2+^ entry explains the “oscillatory” changes in intracellular levels of the ion whereas, the larger scale intracellular Ca^2+^ modulations are attributed to TRPC1-STIM1 associated influx. Physiologically, ICRAC activates proteins like calcineurin that promote translocation of NFAT and subsequent gene expression that is distinct from activation of NFκB based transcription triggered by SOCIC complex (ORAI-STIM1 + TRPC1-STIM1)-mediated ISOC.

**Figure 8:**
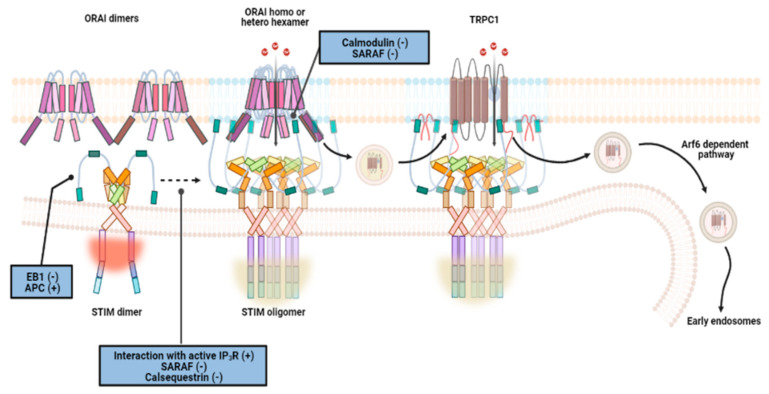
Store-operated Ca^2+^ entry with TRPC1. Per the store-operated Ca^2+^ influx complex (SOCIC) model, Ca^2+^ influx mediated via STIM-ORAI complex has oscillatory patterns whereas bulk cellular entry of Ca^2+^ ions is mediated via TRPC1-STIM1 complex [122,124,146]. As described in the model, depletion of Ca^2+^ ions from the luminal space of the endoplasmic reticulum promotes oligomerization of STIM dimers on ER membrane [105,142,147,148]. Various STIM interacting proteins regulate this process including SARAF, calsequestrin, and activated IP3R. The symbol opposite each of these proteins in the figure depicts the outcome of their interaction with STIM on its oligomerization. As regulators of STIM insertion in the ER membrane, EB1 and APC indirectly influence STIM-ORAI puncta formation as well [136]. ORAI subunits also translocate from low lipid regions (yellow) on the plasma membrane to cluster into hexamers within cholesterol-rich lipid rafts (green). The ORAI-STIM puncta formation allows Ca^2+^ entry while simultaneously triggering detachment of TRPC1 from caveolae in vesicles and insertion into lipid-rich rafts on the plasma membrane [139,141]. From thereon, functional coupling between TRPC1 and STIM brings a large intracellular flow of Ca^2+^ ions. Activated ORAI hexamer also is purported to promote TRPC1 insertion into lipid rafts. After SOCIC-mediated Ca^2+^ ion influx, Rab4 coated vesicles transport TRPC1 via the Arf6 pathway to early endosomes for recycling [140,141]. APC, adenomatous polyposis coli; Arf6, ADP ribosylation factor 6; EB1, microtubule plus end-binding protein; SARAF, SOCE-associated regulatory factor.

Knockdown and mutation analyses reveal that the TRPC1-mediated store-operated Ca^2+^ currents are very much dependent on the expression and normal function of both STIM1 and ORAI1 [141]. Scaffolding proteins (example: Homer1), junction stabilizing proteins (example: Junctate, junctophilins (JP), and extended synaptotagmins (E-Syts)), vesicle-membrane fusion protein (example: synaptosome-associated protein (SNAP-25)), and STIM1 inhibitor protein called SARAF (store-operated Ca^2+^ entry (SOCE)-associated regulatory factor) also regulate TRPC1-mediated store-operated Ca^2+^ entry [142,143]. Some SOCIC modulators directly impact ORAI1 and STIM1 function too. For instance, Homer1 (cytoplasmic) and junctate proteins coimmunoprecipitate and apparently interact with both ORAI and STIM1, thereby promoting the SOCE complex formation [139]. Similarly, CLCA2 or human chloride channel accessory protein 2, a putative tumor suppressor expressed on the cell surface known to enhance SOCE response, colocalizes and coimmunoprecipitates with ORAI1 and STIM1 [144]. STIMATE (STIM-Activating Enhancer) or TMEM110, an ER-resident protein that colocalizes with STIM1 positively impacts SOCE in a two-prong manner. It modulates STIM1-ORAI1 mediated Ca^2+^ signaling by promoting STIM1 translocation to ER-PM junctions and via stabilizing the puncta [145,146]. By contrast, proteins such as CaM (cytoplasmic) and SARAF (ER membrane) directly interact with the ORAI1-STIM1 complex that causes suppression of SOCE response by ORAI1 inactivation (both) and deoligomerization of STIM1 (SARAF only) [141]. The revised SOCIC-based current model for SOCE is thus, visualized, to begin with, ER store depletion that results in the release of STIM1 dimers from the impact of regulators like SARAF and calsequestrin [141,147,148] (Figure 8). As the “free” STIM1 dimers oligomerize, ORAI1 molecules cluster into homo or hetero hexamers in plasma membrane domains that are low in PIP_2_ and cholesterol. ORAI1 clustering triggers the recruitment of TRPC1 tetramers from proximal vesicles into the lipid raft domains. In coordination with the ER-PM junctions coming closer and being stabilized by the tethering proteins (such as junctates, junctophilins (JPH3, JPH4), and E-Syt1), the STIM1 oligomers form independent complexes with the ORAI1 and TRPC1 clusters. APC (adenomatous polyposis coli) facilitates this localization of STIM1 into ER-PM puncta by dissociating the ER Ca^2+^ sensor from microtubule end-tracking protein, EB1. Subsequently, the ORAI1-STIM1 and TRPC1-STIM1 complexes move into cholesterol and PIP2 rich domains to activate ICRAC and ISOC for replenishing Ca^2+^ stores.

#### 2.3.2. Mitochondria and Acidic Vesicles (Mainly Lysosomes)

Mitochondria, known to be the “powerhouse of the cell”, also play a critical role in maintaining Ca^2+^ ion levels in the cytosol and endoplasmic reticulum [83,149,150]. These sphero-cylindrical organelles that are found mostly aggregated around the nucleus store similar levels of intracellular Ca^2+^ as the cytosol (0.1 μM) [140]. Electrochemical proton gradient or membrane potential (~Ψmt = −150 to −180 mV) and close apposition to ER are the two key factors responsible for Ca^2+^ uptake in mitochondria [151]. The free movement of small molecules (less than 5 kDa) from the outer mitochondrial membrane (OMM) into the inner mitochondrial space and their impermeability across the latter generates a high electrochemical proton gradient for ATP synthesis [152]. This gradient simultaneously draws Ca^2+^ ions from the cytosol.

**Figure 9:**
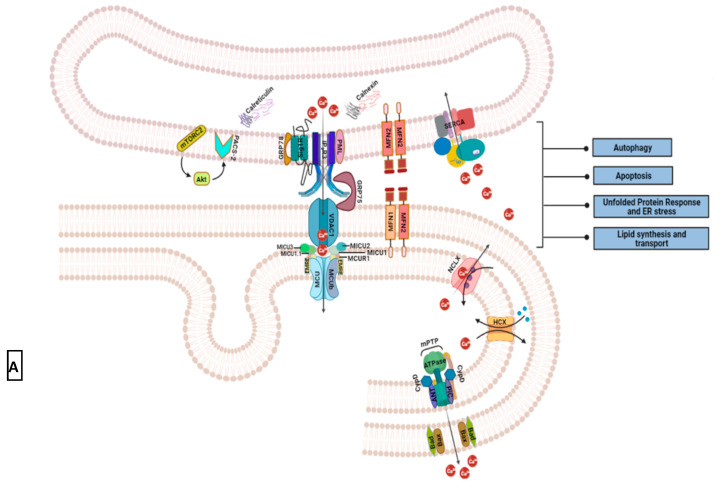

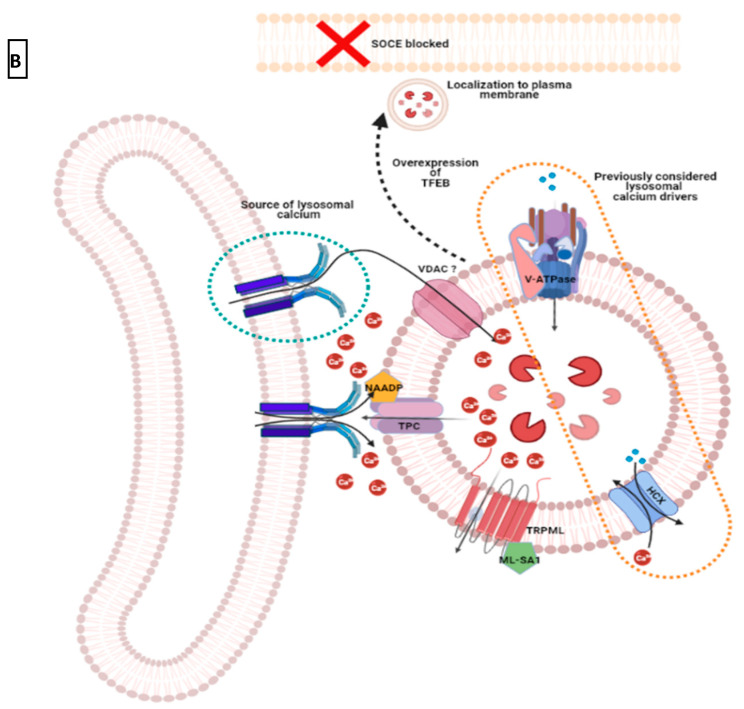
Mitochondrial and lysosomal impact on intracellular Ca^2+^ signal. (**A**) Primary components of Ca^2+^ signaling at the mitochondrial associated membranes (MAMs) include IP_3_R3 on the endoplasmic reticulum, VDAC1 on the outer mitochondrial membrane, and MCU complex on the inner mitochondrial membrane [151,154,156,161]. Transport of Ca^2+^ ions from ER to mitochondria plays a crucial role in cellular metabolism (autophagy), cell survival (during unfolded protein response and impinging cell death signals), lipid production, and distribution [162,163,164,165,166]. For these cellular processes to proceed normally, the integrity of MAMs is essential. Various tethering proteins regulate MAM structural integrity. Such secondary MAM components include GRP75, PML, GRP78 (or BiP), mitofusins (MFN1 and 2), and phosphofurin acidic cluster sorting protein (PACS2) [158,159,160,164,167,168,169]. Localized to the ER side of MAMs, growth factor-activated mTORC2 utilizes phosphorylated PACS2 (via Akt) to help avoid structural disruption of this sub-compartment [170]. It also phosphorylates IP3R3 to promote Ca^2+^ release at MAM sites. Other key MAM localized proteins maintain Ca^2+^ homeostasis in the region. These are calreticulin and calmodulin in the ER lumen, and SERCA on the ER membrane (184). Excessive Ca^2+^ ions in the mitochondrial matrix are extruded mainly by a permeability transition pore (mPTP). This Ca^2+^ efflux pore is situated on the inner mitochondrial membrane and is comprised of F1F0 ATPase with cyclophilin D (CyPD), adenine nucleotide translocase (ANT), and mitochondrial phosphate carrier (PiC) as its regulators [171,172]. Proapoptotic molecules Bax and Bak influence mPTP opening by controlling outer mitochondrial membrane permeability [173]. Ca^2+^ exchangers such as NCLX and HCX are less understood efflux mechanisms that may prevent overload of these cations in the organelle [151,174]; (**B**) Lysosomal Ca^2+^ promotes IP_3_R3 mediated Ca^2+^ release from the endoplasmic reticulum [175,176]. Activated by NAADP, two-pore channels (TPCs) and TRPMLs are the main mode of Ca^2+^ extrusion from lysosomes. Multi-subunit V-ATPases actively transport hydrogen ions into the lysosomal lumen to maintain acidic pH that has been previously considered as the driving force for Ca^2+^ entry into lysosomes via HCX. Although any direct association between acidic lumen and Ca^2+^ store maintenance is now considered controversial, any indirect impact on lysosomal Ca^2+^ stores has not been ruled out. Parallel to this, the dependence of lysosomal Ca^2+^ uptake on ER Ca^2+^ stores is speculated [177]. Lysosomes can also play a more active role in intracellular Ca^2+^ homeostasis as indicated by their inhibition of SOCE [178]. Akt, a serine-threonine kinase; BiP, binding immunoglobulin protein; CyPD, cyclophilin D or peptidyl-prolyl cis-trans isomerase D; EMRE, essential mitochondria regulator; GRP75, glucose-regulated protein 75; HCX, hydrogen-Ca^2+^ exchanger; MCU; mitochondrial Ca^2+^ uniporter; MCUR1, mitochondrial Ca^2+^ uniporter regulator 1; MICU1/2, mitochondrial Ca^2+^ uptake 1 or 2; MFN1/2, mitofusin 1 or 2; mPTP, mitochondrial permeability transition pore; ML-SA1, mucolipin synthetic agonist 1; mTORC2, mTOR complex 2; NAADP, nicotinic acid adenine dinucleotide phosphate; NCLX, mitochondrial sodium Ca^2+^ exchanger; PML, promyeloctic leukemia protein; PACS2, phosphofurin acidic cluster sorting protein 2; SERCA, sarcoendoplasmic reticulum Ca^2+^ ATPase; Sig1R, sigma 1 receptor; TFEB, transcription factor EB; TPC, two-pore channel; TRPML, transient receptor potential mucolipin channel; V-ATPase, vacuolar ATPase; VDAC, voltage-dependent anion channel.

Transfer of Ca^2+^ ions from ER to mitochondria occurs at specialized microdomains or contact sites known as Mitochondrial Associated Membranes (MAMs). These are characterized by the ER and OMM apposed at 10–25 nm from each other and are strewn with a cluster of channels, transporters, exchangers, and tethering proteins for facilitating Ca^2+^ ion transfer [151,152,153,154]. IP3Rs localized at the ER side of the MAMs release Ca^2+^ ions that gate voltage-dependent anion channels (VDACs) located on the OMM [149,153]; Figure 9A. VDACs (1, 2, and 3) are 30 kDa polypeptides having a 19-strand beta-barrel structure that regulates the flux of metabolites (polyvalent anions like ADP and ATP) across the outer mitochondria membranes [155]. These channels transport cations including Ca^2+^ more readily than anions like chloride. Due to voltage-dependent electrostatic gating, the ion selectivity and flux across VDACs change between open and closed states. For instance, the movement of alpha-helix positive charge to the channel outer walls in the closed state increases Ca^2+^ ion flux by 10 times relative to the open state. The importance of proximity between IP3Rs and VDACs in MAMs became clearer when it was realized that the channels on the inner mitochondrial membrane (IMM) transporting Ca^2+^ ions into the matrix have a low affinity (Km ~5–10 mM, KD ~10–50 mM) to these cations [151,156]. These channels, known as Mitochondrial Ca^2+^ Uniporters (MCUs), are highly selective for Ca^2+^ ions, and their opening demonstrates sigmoidal dependence on the cation concentration partly due to lowering of Ψmt that subsequently diminishes drive for cation flux. MCU (40 kDa protein) oligomers form a functional multi-protein complex with their regulators—the mitochondria Ca^2+^ uptake proteins (MICU1, 2, 3) and the essential MCU regulators (EMRE). MICU1 or CBARA1 and MICU2 form obligate heterodimers together in IMM to regulate MCU. These proteins have EF-hands that sense Ca^2+^ ions concentration in the IMS and accordingly inhibit or promote MCU activity. MICU1 is known to stimulate the rapid agonist-mediated Ca^2+^ ions uptake while MICU2 acts as a gatekeeper for MCU during low Ca^2+^ ion concentrations.

MAMs are stabilized by several chaperones and tethering proteins for optimal functioning of key Ca^2+^ ion transporting components, IP3R-VDAC-MCU/MICU1/MICU2 [157,158] (Figure 9A). Mitofusin 1 and 2 (MFN1/2) promote and regulate ER-mitochondria connectivity at MAMs by maintaining ER shape along with the interaction between adjacent mitochondria. Out of the OMM-linked GTPases, MFN2 is directly involved in MAM formation and tends to cluster more in the microdomain via either homotypic or heterotypic (with mitostatin or THCP) interactions, whereas MFN1 has a dominant role in mitochondrial fusion [159]. GRP75, a member of the heat shock protein 70 family (glucose-regulated protein 75 or HSPA9 or mortalin), is an essential cytosolic tethering protein that stabilizes the interaction between IP3R N-terminus and VDAC by acting as a bridge [160]. PML (or pro-myelocytic leukemia), a tumor-suppressor protein enriched on the ER side of MAM microdomains, modulates Ca^2+^ ion release from IP3R by forming a multi-protein complex with the receptor, AKT (or protein kinase B), and protein phosphatase A, thereby modifying the ER-mitochondria Ca^2+^ ion transfer [158]. MAMs are also enriched with ER chaperone proteins like Sigma 1 Receptor (Sig1R) and BiP (immunoglobulin heavy chain binding protein or GRP78) that interact with each other under normal cytosolic Ca^2+^ ion levels. However, with the release of ER Ca^2+^ ions, SigR1 dissociates from BiP to bind and prevent IP3R degradation, thus enhancing Ca^2+^ transfer to mitochondria. 

**Physiological relevance:** Mitochondrial-associated membranes are not just linkage points for ER and mitochondria. These sites finely regulate the movement of ions, metabolites (including Reactive Oxygen Species and lipids), and signaling molecules between the two organelles and thus, are central to normal ER function and mitochondrial biogenesis [164]. Note that triggers for vital processes such as autophagy and apoptosis hinge on the functioning of these dynamic protein bridges that maintain Ca^2+^ ion flow between the two organelles. For example, it is evident from certain studies that post-metabolic stress, autophagy-inducing proteins (like Beclin 1) and the ones involved in autophagosome formation (Atg 14L and Atg5) localize at MAMs. Moreover, the knockdown of MAM complex proteins such as mitofusins impairs autophagosome formation.

Ca^2+^ extrusion is as important as uptake for homeostasis in mitochondria. Two Ca^2+^ exchangers that localize to IMM, a sodium-Ca^2+^-lithium exchanger (NCLX) and a hydrogen-Ca^2+^ exchanger (mCHE) [151,174]. NCLX or SLC24A6 (solute carrier family 24 member 6) is an isoform of plasma membrane sodium-Ca^2+^ exchanger and is mainly expressed in excitable cells [179,180]. It is assumed to exchange three Na^+^ inside the matrix for one Ca^2+^ ion and its electrogenic activity (Ψmt dependent) is inhibited by a selective inhibitor (CGP-37157). Much less is known about the hydrogen-Ca^2+^ exchanger that extrudes one Ca^2+^ ion per 2-3 hydrogen ions [159]. Mitochondrial permeability transition pore (PTP; 3 nm pore diameter), a high conductance non-selective ion channel also has a considerable role in mitochondrial Ca^2+^ ion efflux [181,182]. The remarkable difference in Vmax (maximum rate of reaction) of MCU and the combined efflux rate of Ca^2+^ exchangers creates a huge kinetic imbalance that predisposes mitochondria toward Ca^2+^ ion overload [181]. Opening of PTP prevents this overload via fast Ca^2+^ ion efflux leading to depolarization and subsequent increase in permeability of the inner mitochondrial membrane. The open state of PTP is favored by the presence of Ca^2+^ ions, reactive oxygen species (ROS), mitochondrial matrix pH (around 7.4), and cyclophilin D (CypD) [183]. Inhibitors of PTP include divalent ions like Mg^2+^, Sr^2+^, Mn^2+^, cyclosporin (directly inhibits cyclophilin D), nucleotides, and matrix acidification [181]. The quest to molecularly identify PTP led to the discovery of it being formed by F_0_F_1_ ATPase dimers, though the exact location of the pore within the dimer remains to be identified [184]. PTP is active under normal physiological conditions as well where it functions in a transient low conductance mode to maintain cytosolic Ca^2+^ level without irreversibly changing Ψmt [171]. The positioning of these sphero-cylindrical organelles within the cell, too impacts cytosolic and mitochondrial Ca^2+^ buffering. For instance, the rise in cytosolic Ca^2+^ is limited to the apical or secretory side of pancreatic acinar cells by a “belt” of mitochondria unless the organellar Ca^2+^ buffering capacity is superseded [185].

While IP3 acts as the dominant Ca^2+^-mobilizing messenger, cADPR (cyclic ADP-ribose) and NAADP (nicotinic acid adenine dinucleotide phosphate) are also known to modulate intracellular Ca^2+^ stores [186]. cADPR evokes Ca^2+^ ion release from ER by acting on ryanodine receptors (RyR; counterpart of IP3R in myocytes and co-expressed in some other cell types). NAADP releases Ca^2+^ from acidic and/or secretory vesicles such as lysosomes and endosomes [187]. Although the exact stimulus for intracellular NAADP synthesis has not been established, some studies link activation of certain GPCRs and tyrosine kinase receptors to the formation of this Ca^2+^ messenger.

In most mammalian cells, lysosomes comprise ~5 percent of the cell volume and store similar levels of intracellular Ca^2+^ (0.5 mM) as the ER [175,188] Due to relatively smaller size than ER, lysosomes release nearly undetectable amounts of intracellular Ca^2+^ in response to NAADP trigger [175]. It is hypothesized that this weak Ca^2+^ ion signal is subsequently amplified by ER Ca^2+^ release. Such a model of anterograde Ca^2+^ signal coupling can work only when ER and lysosomes are adjacent. Two-pore channels (TPCs; TPC1 in both lysosomes and endosomes; TPC2 only in endosomes) and TRPML are the prime modes for Ca^2+^ ion release (others include P2X4, VGCCs, TRPA1, and TRPM2) while V-type H^+^ ATPase and H^+^/Ca^2+^ exchanger may concertedly transport Ca^2+^ ions into lysosomes [175,176] (Figure 9B). Some studies support that anterograde ER-lysosomal coupling happens at specialized membrane contact sites (MCS) and have experimentally demonstrated how the removal of TPCs from such sites disrupts the NAADP-based inter-organelle signaling [189,190]. Nonetheless, without a clear understanding of the MCS structure, its functional significance is difficult to verify in anterograde ER-lysosomal Ca^2+^ coupling. Absent the anterograde Ca^2+^ release, it is suggested that lysosomes can participate in refining the ER Ca^2+^ release signal by sequestering Ca^2+^ ions in the MCS microdomain and inactivating background IP3R activity [170,175]. Few cell types are predisposed to retrograde or reverse Ca^2+^ ion signal coupling between ER and lysosomes where IP3 triggers ER Ca^2+^ release that eventually stimulates NAADP synthesis and lysosomal Ca^2+^ ion release via TPCs [191] (Figure 9B). Research is still underway to determine why some cell types have this form of ER-lysosomal signaling and what its physiological relevance is.

## 3. Redistribution of Intracellular Ca^2+^ and Hijack of Its Regulatory Machinery in Cancer Cells

The relationship between Ca^2+^ and carcinogenesis goes back to the 1946 pioneer study by Carruthers et al. where [Ca^2+^]_i_ in mouse and human squamous cell carcinoma was measured to be 57 percent and 47 percent lower, respectively, than respective normal epidermises [192]. Following research further highlighted reduced cellular Ca^2+^ dependency with an increasing degree of neoplastic transformation [193,194,195,196,197,198,199]. Separately, some studies consistently showed how a reduction in extracellular Ca^2+^ beyond a certain point accelerated cellular transformation [193,200,201,202]. Moreover, reduction in cell adhesiveness, enhancement in cell motility, and increased leakage of proteolytic enzymes in cancer cells have been attributed to the loss of cellular Ca^2+^ [203,204,205]. To complement the in vitro findings, various epidemiological studies have yielded evidence for the inverse correlation between intake of dietary or supplementary Ca^2+^ and cancers of colon, rectum, breast, gastric tract, endometrium, renal system, and ovaries [206]. Although the above-mentioned evidence on the differential levels of Ca^2+^ ion between normal and tumor cells would suggest this cation to have a tumor-inhibitory effect, newer studies indicate a rather complex dynamic [207,208,209]. In this section, we will bring to light multiple ways in which cancer cells manipulate intracellular [Ca^2+^]_i_ levels and the associated molecular machinery during different stages of tumor progression. Each subsection will provide an overview of studies supporting distortion of signaling in a specific intracellular Ca^2+^ pool, thereby explaining the mechanisms underlying various cancer hallmarks—excessive proliferation, inhibition of growth suppressors, activation of invasion and metastasis, replicative immortality, induction of angiogenesis, resistance to cell death, dysregulated cellular metabolism, and immune surveillance evasion [210]. Additionally, the tables summarize the role of individual Ca^2+^ signaling components described earlier during each of the hallmark cellular processes involved in tumorigenesis and cancer progression. They also provide a corresponding list of potential therapeutic drugs targeting Ca^2+^ signaling proteins under preclinical conditions.

### 3.1. Intracellular Ca^2+^ Pool in the Endoplasmic Reticulum and at the ER-PM Junction

Because of the substantial observed association of aberrant expression or dysfunction of ion channels with tumor initiation and progression, cancer is at times termed as “oncochannelopathy” [211]. Multiple Ca^2+^ channels expressed on the plasma membrane are functionally altered in cancer cells [210,211,212,213] including voltage-gated Ca^2+^ channels (VGCCs), ligand-gated channels, TRP channels, and ORAI channels. Bioinformatic analysis has revealed abundant expression of genes encoding most VGCCs (L-, R-, N-, P/Q-, and T-type) in cancer tissues [214,215,216,217,218]. Accordingly, CACNA1G encoding the pore-forming alpha subunit of T-type VGCC, Cav 3.1, is expressed in lung adenocarcinoma (A549), colon cancer (HCT116), breast cancer (MCF-7, MDA-MB-231), ovarian cancer (A2780), and melanoma [208,215,217,218,219,220,221,222]. Similarly, Cav 1.3 (CACNA1D encoded L-type VGCC) is shown to be upregulated in breast, endometrial, prostate, neuroblastoma, and colorectal cancer biopsies [217,223]. Consequently, blocking VGCC activity either chemically (BK10040 and KYSO5090; T-type channel blockers or Verapamil; L-type blocker) or by gene silencing is reported to inhibit growth, induce death, or reduce migration of tumor cells by causing a drop in intracellular Ca^2+^ [217,223,224,225,226,227].

The somewhat controvertible, but the generally accepted, role of ligand-gated Ca^2+^ channels in cancer cells is anti-inflammatory/protumorigenic for P1 receptors and proinflammatory/anti-tumorigenic for P2 [228,229,230]. Due to higher levels of ATP in tumors than normal tissues, purinergic receptors, P2X and P2Y, are the most extensively studied ligand-gated Ca^2+^ channels in tumors [231]. ATP binding to P2X receptors results in increased Ca^2+^ influx through them while P2Y receptor (G-protein coupled) activation by the nucleotide enhances ER Ca^2+^ release or cAMP production [231,232]. The receptor expression levels and related Ca^2+^ signaling are both altered during growth factor-induced epithelial–mesenchymal transition (EMT) [232,233]. For instance, EGF-mediated EMT in MDA-MB-468 breast cancer cells is reported to reduce the sensitivity of P2Rs to ATP binding with simultaneous downregulation of P2Y13 and upregulation of P2X5 and P2Y6 transcript levels. ATP-mediated increase in [Ca^2+^]_i_ can exert anti-migratory and antiangiogenic effects on tumor-derived endothelial cells. Two separate studies have shown that treatment of Breast Tumor-derived Endothelial cells (BTEC) and Renal Tumor-derived Endothelial Cells (RTEC) with 100 mM ATP leads to a biphasic increase in [Ca^2+^]_i_—an initial transient release from ER followed by SOCE and then a prolonged release rise due to Ca^2+^ influx via P2X7R and P2Y11R [234,235]. This raised [Ca^2+^]_i_ then activates adenylate cyclase10/cAMP/EPAC-1 pathway to disrupt actin cytoskeleton and thus, inhibit migration of tumor-derived endothelial cells.

The modification of Ca^2+^ ion efflux machinery at the ER-PM junction during tumor formation or progression also requires attention. PMCA and Na^+^/Ca^2+^ Exchanger (NCX) lead a concerted effort on a normal cell surface to expel excessive free cytosolic Ca^2+^ ions and thereby prevent cytotoxicity [236]. Functionally, it is logical to consider deactivation or negligible expression of the efflux machinery in cancer cells so that high intracellular Ca^2+^ is available to drive tumor proliferation and metabolism [41,237,238]. Such has been observed in colon cancer where PMCA4 mRNA levels were found to be significantly lower in high-grade colon adenocarcinoma, lymph node metastasis, and benign tumors relative to healthy tissue [36,239,240]. Using the HT29 colon cancer cell line, Aung et al. showed that PMCA4 overexpression indeed minimizes cell proliferation in colon cancer [239]. Moreover, PMCA4 expression is observed to be induced during differentiation of colon cancer (HT29), neuroblastoma (IMR-32), and breast cancer (MCF7) [36,241,242,243]. By contrast, pancreatic cancer cells that exhibit high glycolytic over mitochondrial metabolism need PMCA for cell survival [244]. PMCA4 function in these PDAC (pancreatic duct adenocarcinoma) cell lines relies on ATP derived from glycolysis. Therefore, inhibiting ATP production by disrupting glycolysis stops PMCA driven Ca^2+^ ion efflux resulting in excessive Ca^2+^ filling and cell death [245]. Expression of PMCA4 mRNA is muted in breast cancer cell lines too. However, expression of its splice variant PMCA4b is complex due to distinctive regulation by histone deacetylase inhibitors and ERα [243,245,246]. As an example, PMCA4b exhibits low expression with high sensitivity to HDAC inhibitors in MCF-7 (ER-positive cell line), but high expression and low sensitivity to 17β estradiol or HDAC inhibitors in MDA-MB-231 cells (triple-negative breast cancer) [245]. Kenealey et al. have also shown that inhibition of PMCA activity (likely PMCA1 and 4) by resveratrol induces programmed cell death in MDA-MB-231 cells [241].

The Ca^2+^ antiporter NCX1 is the most widely distributed and well-studied isoform within its family. Its mRNA and protein expression levels are dramatically reduced in renal cancer cells and nephroblastoma, conferring several advantages [247]. Knockdown of NCX1 in MDCK cells induces EMT via the Ca^2+^-dependent ERK signaling activation. However, the ability of NCX1-knockdown kidney cells to grow in an anchorage-independent manner and their increased junctional permeability is independent of the Ca^2+^ transport function of the exchanger [248]. In penile tumors, knockdown of NCX1 lifts the brakes on proliferation and reduces apoptosis [247,249]. For therapy-resistant medulloblastoma and ovarian cancer, on the other hand, knockdown of NCX1 results in sensitization of these cancers to ionizing radiation and cisplatin [250,251]. Similarly, OSW-1, a natural saponin, and potential anticancer treatment blocks NCX activity in acute leukemia cell line (HL-60) and induces cytotoxicity via accumulation of excess Ca^2+^ ion in the cytosol [252]. In cancer cells exposed to hypoxia, the reverse mode NCX functioning is coupled to carbonic anhydrase IX (CA1X) and sodium-hydrogen exchanger (NHE1) for converting the intracellular proton load (occurring due to metabolic changes during hypoxia) into interstitial acidosis [253,254]. This allows the breakdown of the extracellular matrix and consequently, promotes tumor cell migration and invasion [253,255].

SOCE is central to intracellular Ca^2+^ signaling as it forms the major Ca^2+^ ion influx route in non-excitable cells [256]. Conceivably, the expression and activity of SOCE components are remodeled during each stage of cancer progression. However, the pattern of SOCE alteration for advancing cancer hallmarks is complex because it is based on the cancer cell type, progression stage, and isoforms of participating components [257,258,259]. As discussed earlier, STIM1 and ORAI1 are the “classical SOC” channel components with STIM2, ORAI2, and ORAI3 taking the center stage in selective cases for SOCE-mediated Ca^2+^ influx [257,260]. Evidence from pharmacological and molecular inhibition studies underscores the role of SOCE in cell cycle progression [257,261,262,263,264,265]. Store-operated Ca^2+^ entry is elevated during G1/S transition but decreased during entry into the G2/M phase [266]. Diminishing SOCE via STIM1 inhibited the proliferation of cervical cancer, glioblastoma (U251), osteosarcoma (143B and U2OS), lung carcinoma (A549 and SK-MES-1), and breast cancer (MCF7) [265,266,267,268,269,270,271,272]. Knockdown of STIM1 in cervical cancer (SiHa and HeLa) caused inhibition of CDK2 phosphorylation, increase in cyclin inhibitors p21 and p27, and accumulation of Cyclin E [272]. In hepatoma cell lines (Huh-7 and HepG2), simultaneous knockdown of STIM1 and ORAI1 dropped protein levels of cyclin D1, thus causing G0/G1 cell cycle arrest [272]. Various studies have also shown that ORAI3-actuated SOCE in MCF7 upregulates c-myc/NFAT/p-ERK axis to increase in CDK2/4, cyclins D1 and E, and promote G1/S transition [266,273,274]. Interestingly, STIM2 upregulation in melanoma cells has been known to contribute to antiproliferative but invasive phenotype [275]. Modulation of SOCE components and the associated Ca^2+^ uptake by cancer cells also influences programmed cell death. Based on the cell type and stimuli, SOCE can either aid apoptosis or provide apoptotic resistance in cancer cells [276,277,278,279,280]. The pivotal proapoptotic role of STIM1/ORAI1 was first noted in pancreatic cancer where downregulation of ORAI1 or ectopic expression of its dominant-negative mutant reduced the susceptibility of tumor cells to apoptotic stimuli [280]. On the other hand, overexpression of ORAI1 in androgen-independent prostate cancer cells can reinstate the normal level of apoptosis. The proapoptotic role of STIM1/ORAI1-based SOCE is reversed in non-small cell lung cancer (A549), ovary carcinoma (A2780), pancreatic adenocarcinoma (HC67, Panc1, Capan1, ASPC1, and MiaPaca2), multiple myeloma (immortalized human cell lines and patient-derived tumor cells), and melanoma (B16BL6-8) with the general accompaniment of elevated Akt pathway activity [277,281,282,283,284,285]. ORAI1-based Ca^2+^ entry also slows down the rate of CD95 mediated apoptosis in leukemic T cells [286]. Moreover, STIM2, found abundantly in colon cancer cells, can elicit an anti-apoptotic effect on such tumor cells [287]. Likewise, ORAI3 overexpression in ER-positive T47D cell line confers apoptotic resistance to cisplatin, paclitaxel, or other chemotherapeutics [288].

Unlike apoptosis, all STIM and ORAI isoforms exert a uniform positive effect on tumor migration and invasiveness [276]. Studies on human cervical cancer have revealed that patients with STIM1 upregulated primary tumors have poorer clinical outcomes due to excessive tumors and lymph node metastasis [257,289]. Similarly, STIM1/ORAI1 overexpression in multiple myeloma or upregulation of ORAI1 in esophageal squamous cell carcinoma is associated with poorer progression-free survival [257,269,284]. One of the pioneer studies demonstrating the proinvasive role of STIM1/ORAI1 in breast cancer cells determined that SOCE enhanced turnover of Rac- and Ras-based focal adhesions to increase cancer cell migration [257,290]. A similar role for STIM1/ORAI mediated SOCE has been observed in cervical cancer, hepatocellular carcinoma, renal cell carcinoma, nasopharyngeal cancer, and glioblastoma where this Ca^2+^ influx regulates focal adhesion turnover, cytoskeletal reorganization, and actomyosin-based mechanotransduction [291,292]. Except for melanoma, STIM2 overexpression in cancer cells and their invasiveness are inversely correlated [284,293]. Understandably, therefore, a high STIM1/STIM2 ratio in breast cancer cells combined with increased SOCE correlates with poorer prognosis in patients [276,284].

Increased angiogenesis is another quintessential feature of cancer [210]. Therefore, manipulation of SOCE has been carefully studied in cancer-associated stromal cells, especially endothelial cells [294]. Silencing of STIM1 or ORAI1 in vascular endothelial cells (HUVECs) attenuates cell proliferation and VEGF-triggered Ca^2+^ influx [276,295]. On the other hand, STIM1 in vivo overexpression in cancer cells positively correlates with increased VEGF secretion, endothelial cell proliferation, and thus angiogenesis [296]. Studies have also shown ORAI1 to stimulate in vitro tubulogenesis and in vivo angiogenesis [269]. Immune cells are another integral and active part of the tumor microenvironment. Since SOCE components were first discovered and studied in T cells, it is presumable that their function in cancer-promoting or -killing immune cells has been extensively explored [297,298]. Surprisingly, distortion of the role of SOCE components and thereby, the intracellular Ca^2+^ flux in cancer-related T cells is not well investigated. One of the early studies in this area delineated how downregulation or absence of STIM1 and STIM2 in cytotoxic T lymphocytes (CTLs; a subset of anti-tumor immune cells) prevents the production of cytolytic factors (interferon-γ and TNF-α), thus releasing control on tumor cell engraftment and in vivo tumor growth [299]. Contradictorily, partial inhibition of ORAI1 in a separate study resulted in the killing of CTLs by tumor cells; more research is, therefore, warranted [300].

IP3Rs, as the principal Ca^2+^ ion release channels for endoplasmic reticulum, are found localized at various sites on the ER membrane: near the SOCE nanodomain, the ER-PM junction outside of the nanodomain, and at the mitochondrial-associated membranes (MAMs) [301,302,303,304]. The functionality of these receptors is determined by their localization and isoform type. Receptors expressed in the ER-PM junction participate in the initiation and propagation of SOCE, whereas the receptors expressed at MAMs regulate ER-mitochondrial Ca^2+^ ion movement, thereby influencing cellular bioenergetics and apoptosis [165]. As our focus here is on the ER-PM junction, the role of IP3Rs at MAMs will be discussed in the latter part of this article.

IP3Rs (and the smooth muscle counterparts, RyRs) control cellular functions like contractility, cellular motility, and migration [305]. Although the research on the role of IP3Rs in cell migration is still new, studies, in general, support a promigratory effect [305,306]. A migrating cell has polarized front and rear ends that differ in the cytoskeletal arrangement and Ca^2+^ ion gradient. The repetitive attachment-detachment of leading and trailing edges during cell migration are accompanied by Ca^2+^ ion oscillations. As a result, migrating tumor cells should have a strong dependency on Ca^2+^ channels [307,308,309]. This hypothesis is validated by Baljinnyam et al. via their study demonstrating that the activation of PLCε/IP3/IP3R in melanoma cell lines (Mel-2 and SK-Mel-24) increases cell migration; the resulting Ca^2+^ ion flux promotes interaction between S100A4 (Ca^2+^-activated invasion protein) and myosin light chain kinase II which rearranges the actin cytoskeleton via EPAC/cAMP-induced Ca^2+^ elevation [305,310]. IP3R3 as well has been found to cause peritoneal dissemination of gastric cancer [305,311]. Ano1, or TMEM16A, a Ca^2+^-activated chloride channel (CaCC) at the ER-PM junction, is highly upregulated in several cancer types—gastrointestinal tumors, head and neck squamous carcinoma, glioblastoma, pancreatic, breast, and colorectal cancer— as a pro-proliferative and pro-migratory signaling molecule [312,313,314,315,316,317]. IP3Rs are now known to tether Ano1 to ER-PM junctions, provide localized Ca^2+^ ion for their activation, and link the opening of CaCCs to SOCE augmentation along with GPCR stimulation [318,319].

By maintaining intracellular Ca^2+^ homeostasis, voltage-independent Ca^2+^ channel family known as Transient Receptor Potential or TRP channels influence various Ca^2+^ ion-sensitive downstream effectors of cellular processes like proliferation, motility, and apoptosis [320,321]. TRP channels having a pro-proliferative effect in cancer cells are mostly derived from TRPC (5, 6), TRPM (2, 4, 5, 7, 8), and TRPV (1, 2, 6) subfamilies [322,323]. TRPC5 downregulation reduces growth in adriamycin-resistant breast cancer cells due to concomitant decline in drug resistance imparting P-glycoproteins [324]. TRPV1, TRPV2, and TRPM2 have been observed to stimulate the proliferation of prostate cancer [325]. Moreover, overexpression of TRPV2 or upregulation of TRPV6 positively correlates with poor prognosis of esophageal squamous cancer and malignant transformation in leukemia or other tumors, respectively [322,326,327,328]. Interestingly, TRPV channels trigger cancer cell proliferation based on cellular subtypes. For example, TRPV1 is expressed in several breast cancer cell lines, peculiarly is found to inhibit cellular proliferation in triple-negative breast cancer [329]. Proapoptotic members of TRP channels largely belong to TRPC, TRPM, and TRPV subfamilies [322]. TRPC1 in conjunction with STIM1 has been recently shown to induce cisplatin cytotoxicity in non-small cell lung cancer cells via reactive oxygen species and DNA damage response [280,330]. In addition, TRPC1 and TRPC4 are postulated to sensitize triple-negative breast cancer to various chemotherapeutics [331]. Among the TRPM members, TRPM2 activation with subsequent Ca^2+^ ion elevation causes oxidative stress and apoptotic death in MCF7 and CaCo-2 cell lines treated with 5-fluorouracil and leucovorin [332]. Conversely, TRPM8 protects pancreatic cancer cell lines Panc-1 and BxPC3 from gemcitabine by maintaining the expression of multi-drug resistance proteins like P-glycoprotein [333]. TRPV1 and downstream Ca^2+^ ion signaling get activated in breast cancer MCF7 cells and glioblastoma treated with TRPV1 positive allosteric modulator MRS1477 or capsaicin, respectively, to trigger cell death [334,335]. Members of the TRP family are also involved in mechanotransduction that allows the cancer cell to invade and metastasize [336]. TRPM7 is the best-studied example in this context with its expression in nasopharyngeal and pancreatic cancer related to poor prognosis [337,338]. This non-selective Ca^2+^ ion-permeable channel forms a mechanosensory complex in breast cancer cells [336,339]. Thus, silencing TRPM7 in ER-positive MCF7 and triple-negative breast cancer MDA-MB-231 redistributes filamentous actin in cell cortices, raises the number of focal adhesions due to rearrangement of local Ca^2+^ ion levels, and phosphorylates myosin light chain and paxillin [339]. Amongst the TRPV subfamily, TRPV4 is significantly upregulated during breast cancer metastasis, and in the clinical samples with poor overall survival has been observed [340,341].

By dint of its central role in ER Ca^2+^ replenishment, SERCA pumps are essential for proper protein folding and maturation and other functions required for cancer cell proliferation and survival [342,343]. Therefore, various cancer types such as colon, lung, and prostate carcinomas are rife with mutations and changes in SERCA expression levels (expression datasets of human cancer cell lines) [343,344,345,346,347,348,349]. On the other hand, SERCA3 expression is induced during differentiation but negatively correlated to colon cancer progression (DLD-1, COLO-205, and Caco-2) [345,349]. Following suit, high expression of SERCA3 is observed in differentiated forms of gastric cancer and myeloid leukemias [350,351]. SERCA pumps are also recognized as regulators of Notch1 receptors that are common drivers of tumor proliferation, especially for leukemia [352]. As a result, SERCA inhibition blocks the activity of mutated Notch1 receptors (by impairing protein folding) which leads to G0/G1 arrest in leukemia cells. SERCA isoforms are required even for the survival of cancer stem cells (CSCs) against metabolic stress [353]. In a study utilizing metabolic stress-resistant breast cancer CSCs derived from MCF-7 and MDA-MB-231 cell lines, SERCA expression was induced in a calmodulin kinase 2α (CaMK2α)/NFκB-dependent manner to prevent stress elicited by 2-deoxy-glucose treatment. Treatment of such CSCs with a combination of 2-deoxy-glucose and thapsigargin, however, sensitized the cells to apoptosis via cytosolic Ca^2+^ ion overload. To prevent unnecessary cytotoxicity induced by thapsigargin in normal cells, its soluble prodrug, G-202 or mipsagargin, was designed and tested in a Phase II study against sorafenib-resistant hepatocellular carcinoma (HCC) [354]. In the study, mipsagargin was shown to stabilize tumor progression by specifically targeting PSMA (prostate-specific membrane antigen) expressing endothelial cells that form the HCC-associated vasculature. It has also been tested against glioblastoma, prostate cancer, and renal cell carcinoma; however, results of those trials have not yet been published.

The anomalous signaling, localization, and mobilization of Ca^2+^ ions seen in tumor cells are partly attributed to peculiar expression levels of calmodulin (CaM) and its target proteins [355]. It is a common observation for tumor cells to express higher levels of CaM which complements the raised levels of cytosolic Ca^2+^ ions relative to benign tissues [356,357,358,359,360]. Although it was contended that this observation was cell type-, culture condition-, or transformation agent-specific, Wang et al. (1992) confirmed a significant increase in CaM levels at the individual cell level between transformed and normal cells during G1 to S phase transition [361]. It was further validated that the surge in CaM levels was specific to cellular transformation and not due to a rise in total intracellular protein during cell cycle progression. Inhibition of CaM by the naphthalensulfonamide-derived selective CaM antagonists W-7 and W-13 caused p21cip1-dependent growth arrest and apoptosis of multiple myeloma tumors in a mouse xenograft model [362]. Some of the protumorigenic effects of calmodulin are facilitated through its binding to the Ca^2+/^calmodulin-stimulated protein kinase (CaMK) family that modulate cell cycle progression by interacting with phase-specific cyclins and cyclin-dependent kinases [363]. For instance, CaMKI allows G1/S transition via phosphorylation and activation of cdk4 while CaMKII triggers metaphase to anaphase progression by stimulating cdc2 [364,365,366]. Therefore, pharmacological (STO-609, KN-62, KN-93, berbamine, etc.) and siRNA inhibition of CaMKs have demonstrated anti-proliferative effects in numerous cancer cell lines [367]. Direct interaction between CaM and nuclear hormone receptors is another exploitable mechanism for limiting tumor proliferation. This is exemplified in studies where the growth of estrogen receptor- and androgen receptor-positive cancer cell lines, MCF7 and LnCaP, respectively, is inhibited by CaM antagonists. In such cell lines, CaM antagonists disrupt stabilization and activation of estrogen receptor and androgen receptor, both of which are dependent upon direct interaction with CaM [368,369,370]. The functional interdependencies of calmodulin and numerous [Ca^2+^]_i_ transporters too can be leveraged for killing cancer cells. It is well known that calmodulin regulates the slow inactivation of CRAC currents by direct interaction with the STIM1 SOAR domain, thereby disrupting the STIM1-ORAI1 complex [371]. It has also been established that calmodulin-binding is required for IP3R activation [372]. On the other hand, CaMKII potentiates SOCE by promoting STIM1-ORAI1 complex formation [373]. CaM and CaMKs are not only regulators of SOCE, but also form integral components of its downstream signaling cascade. This is well evident by their activation of Akt, ERK1/2, and Raf/Pyk2 (via cytosolic Ca^2+^ elevation) for tumor survival, growth, migration, and invasion [374,375,376,377,378].

Calreticulin (CRT) and calbindin are other major EF-hand containing Ca^2+^-binding proteins that are altered by cancer cells [379,380,381,382]. Cancer cells gain many advantages over normal tissue by dysregulating calreticulin since it is a critical modulator of Ca^2+^ ion-dependent processes including proliferation, differentiation, cell adhesion, migration, intercellular interactions, immune response, and apoptosis [383,384,385,386,387,388,389,390]. Accordingly, upregulation of CRT expression is recorded in various tumors such as invasive breast carcinoma, high microsatellite instability colorectal carcinoma, squamous cell carcinoma, leukemia, and many more [391,392,393,394,395]. CRT expression also positively correlates with tumor size, grade, and development stage in breast and lung cancers [396,397]. Even more, its high expression levels are commensurate with poor survival in prostate cancer and neuroblastoma patients [398,399]. The effect of CRT on tumor proliferation is cell type-dependent with most cancer cells responding to upregulation of this protein with rapid growth [400,401]. For instance, overexpression of CRT in pancreatic and gastric cancer cells causes remarkable growth of these tumor types while stable knockdown of the same in oral squamous cell carcinoma led to significant G0/G1 arrest with a negative impact on anchorage-independent growth and colony formation. In certain cancers like gastric carcinoma, CRT overexpression is reported to promote VEGF expression as well, thus leading to enhanced angiogenesis, tumor invasiveness, and migration [401]. Under normal conditions, calreticulin is essential for cellular adhesion via integrin-associated Ca^2+^ signaling with the Wnt pathway as the potential downstream effector [402,403]. In contrast, CRT N-terminus expressed on the cancer cell surface is found to be essential for thrombospondin (TSP) mediated invasion and metastasis [404]. Studies have elucidated that the N-terminus of TSP binds to the CRT and low-density lipoprotein receptor co-complex to induce disassembly of focal adhesion kinase, thus reducing cellular adhesion. CRT as a promoter of cancer cell invasion and metastasis has been verified through overexpression (MDCK cells, gastric cancer cell line AGS) and knockdown (HL60 leukemia cell line and J82 bladder cancer cells) studies as well [405,406,407,408]. Strikingly, CRT expressed on the cancer cell surface can engage in immunomodulatory activities and pose a threat to tumor survival. Work from de Bruyn and Bremer et al. revealed that TRAIL-induced apoptosis relocates CRT from ER lumen to cell surface where the Ca^2+^-binding protein is shown to attract dendritic cells and macrophages for phagocytosis [409]. In glioma, an increase in CRT expression likewise correlates with higher radiosensitivity and, thus, a greater rate of apoptosis [410]. In pancreatic adenocarcinoma, cancer cell survival and chemoresistance decrease significantly post CRT knockdown [411]. Very limited research has been conducted on modifications of calbindin activities in cancer cells. This Ca^2+^ ion buffer which has dominant expression in neurons has so far only been found to protect osteosarcoma cells from apoptotic stimuli [412].

### 3.2. Intracellular Ca^2+^ Pool at ER-Mitochondrial Junction

Aberrant cellular metabolism, a hallmark of a wide variety of tumors, stems from the dysfunction of mitochondrial pathways for ATP production [210]. The shift from oxidative phosphorylation to glycolysis for ATP generation in a subset of cancers is induced by the cumulative effect of mutations in the participating mitochondrial enzymes and Ca^2+^ signaling [413,414,415,416]. Specifically, activities of TCA cycle enzymes, alpha-ketoglutarate, isocitrate dehydrogenase, and pyruvate dehydrogenase rely on proper Ca^2+^ ion uptake by mitochondria. Thus, plummeting Ca^2+^ ion transport from ER to mitochondria in transformed cells portends tumor progression.

ER-mitochondria junctions or Mitochondrial-Associated Membranes (MAMs) are hubs for proteins that are integral to vital processes such as phospholipid synthesis and translocation, mitochondrial Ca^2+^ ion transport, ER stress, ER Ca^2+^ release, mitochondrial morphology regulation, cellular bioenergetics, apoptosis and survival, autophagy, and ROS generation [165]. IP3R, as discussed earlier in this review, is one of the MAM proteins involved in ER to mitochondria Ca^2+^ ion release, and thus crucial for autophagy and apoptosis induction [417,418,419]. Simplistically, inhibition of IP3R (mainly IP3R3) mediated Ca^2+^ ionrelease into mitochondria creates an imbalance in ATP/ADP ratio that derepresses AMPK/mTOR/ULK-1 axis and initiates autophagy flux (based on nutrient starvation) [418]. Notably, abrogation of IP3R activity outside of MAMs perturbs autophagy induction independent of mTOR suppression—no binding of IP3R to Beclin1, a protein required for autophagosome formation, is observed [420]. Conversely, IP3Rs located away from MAM regions of the ER can promote autophagy induction by acting as Ca^2+^ ion leak channels as less Ca^2+^ storage in the ER manifests into poor ion supply for ATP production [418]. Depending on the tumor stage and type, autophagy flux can either cause a cancer cell to thrive or perish [421,422]. By and large, autophagy activation supports tumor suppression during the early stages of cancer development whereas, more advanced tumors utilize it for drug resistance and sustained growth. Autophagy induction due to inadequate ATP production in normal cells halts G1/S cell cycle progression because of an increase in p53/p21 expression and activity [423,424,425]. In cancer cells with mutant p53, however, aberrant proliferation continues post autophagy induction that results in mitotic catastrophe and eventually cell death due to deprivation of mitochondrial metabolites [423,424,425,426,427]. Removal of dysfunctional proteins and mitochondria during autophagy can also prevent tumorigenesis [428]. In highly developed tumors and related stem cells, autophagy induction contradictorily promotes survival from chemotherapeutic (such as 5-FU or bortezomib) assault; competition between a plethora of tumor suppressors and promoters or oncoproteins to interact with IP3R is consistent with the above-stated observation [429,430,431]. Within this context, Bax inhibitor-1 (BI-1) and Bcl2, both located on the ER membrane, stymie IP3R mediated Ca^2+^ ion release into the mitochondria, ergo inducing autophagy [432,433,434]. Inversely, tumor suppressors such as Beclin-1 prevent inhibition of IP3R-mediated Ca^2+^ ion release into the mitochondria partly by interacting with Bcl2 which then dissociates from IP3R, thereby restoring the ER-mitochondrial Ca^2+^ ion transfer [420]. Chemotherapeutics like Arsenic trioxide also increase this Ca^2+^ ion transport by upregulating the expression of tumor suppressor PML (promyelocytic leukemia) that blocks phosphorylation and inactivation of IP3R by p-Akt [418,435,436].

The ER-mitochondria Ca^2+^ ion transport has emerged as an integral component of the crosstalk between autophagy and apoptosis [437]. Inhibition of autophagy or excessive mitochondrial Ca^2+^ ion uptake via MAMs triggers intrinsic apoptosis pathways. Thus, tumor suppressors and proapoptotic molecules like BRCA1 or PTEN bind to IP_3_R to boost Ca^2+^ ion uptake at MAMs while tumor promoters and anti-apoptotic molecules such as Bcl2 or Bcl-xL repress mitochondrial Ca^2+^ ion overload by binding to and inhibiting the channel activity [438,439]. VDAC1 and GRP75, members of a trio comprising IP3R and forming a Ca^2+^ ion transporting complex at MAMs are also exploited by cancer cells [440,441]. It is noteworthy that the impact of VDAC1 manipulation on cancer cell bioenergetics and survival stretches beyond its role in the trio [442,443]. Protumorigenic proteins, hexokinase I and II (HKI and HKII) partner with VDAC1 on the outer mitochondrial membrane to utilize its nucleotide shuttling property (ATP/ADP) for generating high energy storage forms like glucose-6-phosphate and fuel rapid cell proliferation via glycolysis [442,444]. Aside from this, Bcl2, Bcl-xL, or hexokinase bind to the N-terminus of VDAC1 and prevent the release of cytochrome C through a VDAC1 multimer pore that assembles in response to apoptotic stimuli [445,446,447]. At the inner mitochondrial membrane, the Ca^2+^ uptake channel, MCU with its regulators MICU1/2 act as the key mediators of Ca^2+^-based mitochondrial functions [448,449]. Because MICU1/2 limits Ca^2+^ ion uptake via MCU and its absence causes cell death via mitochondrial overload, tumor cells are widely observed to have elevated levels of these proteins, although expression of MCU is subtype-dependent [172,450]. For example, in invasive breast carcinoma such as Triple-Negative Breast Cancer, upregulated MCU expression is critical for xenograft size, lymph node metastasis, and lung infiltration [451,452]. On the other hand, downregulation of MCU can negatively impact breast cancer cell size and motility [452].

### 3.3. Intracellular Ca^2+^ Pool at ER-Lysosome Junction

Ca^2+^ signaling at lysosomal membranes and surfaces of acidic vesicles provides a functional scaffolding for endocytic traffic and autophagy, thereby directly influencing cellular health [453,454].

Alteration of such signaling in cancer contributes to cancer hallmarks such as uninhibited growth, angiogenesis, and metastasis [455,456,457]. TPC1 and TPC2 are considered the drivers of lysosomal Ca^2+^ signaling [458]. Initial research in a mouse model of B16 melanoma cells and xenografts established the role of these Ca^2+^ ion release channels in tumor invasiveness [459]. The study found that VEGF treatment caused NAADP-mediated, sustained Ca^2+^ ion signals via TPCs on lysosomal membranes, and this promoted G0/G1 cell cycle transition, tumor vascularization, focal adhesion kinase (FAK) formation, and migration. Both in vitro and in vivo silencing or pharmacological inhibition of TPC1/2 have also been shown to block invasion and migration of urinary bladder carcinoma (T24), hepatoma (HUH7), and mammary carcinoma [189,460]. Nguyen et. al used a mouse mammary carcinoma model and silenced TPC expression to demonstrate reduction in lung metastasis. The delineated mechanism involved a downstream failure in the trafficking of β1-integrin to plasma membrane that further prevented phosphorylation of FAK, Src, and vinculin, and thus lamellipodia formation [460]. This complements the previously established role of lysosomes in enhancing tumor migration via cathepsin-based disintegration of extracellular matrix [189,461].

TRPML1 is another Ca^2+^ ion release channel expressed on lysosomal membranes and is known to finely control autophagy [458,462]. Presently, there is low evidence of its involvement in cancer progression, but the expression of its downstream Ca^2+^ activated transcription factor, TFEB is noticeably correlated with cellular malignancy [189,463,464,465,466]. Nutrient starvation and ROS production can activate TRPML1 mediated Ca^2+^ ion release followed by calcineurin activation that subsequently dephosphorylates TFEB and releases it from 14-3-B-B; free TFEB is then translocated to the nucleus to evoke transcription of autophagy-related genes [467]. In short, cancer cells benefit by mai17ntaining dephosphorylated TFEB levels. Supporting this hypothesis, non-small cell lung carcinoma patients with the poor outcome often have TFEB (dephosphorylated) overexpression with simultaneous upregulation of other lysosomal markers like LAMP2a and cathepsin D [468]. TFEB upregulation is also linked to higher invasiveness in colorectal cancer cells [469]. Although a less known function of TFEB, DNA repair triggered by this protein is exploited by Triple-Negative Breast Cancer cells for chemoresistance against doxorubicin [470].

## 4. Conclusions and Discussion

**Figure 10:**
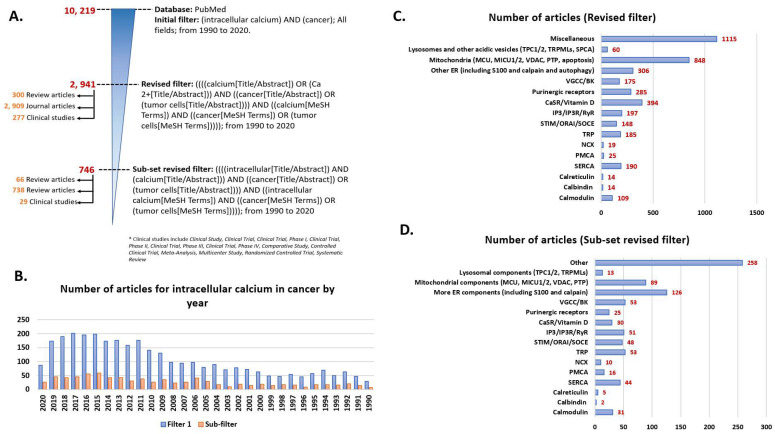
Trends observed in published articles on the role of intracellular Ca^2+^ in cancer. (**A**) Schematic of finding articles published from 1990 to 2020 on intracellular Ca^2+^ in cancer. The number of articles published based on each advanced search filter combination is indicated in red. All the articles found with revised filter and subset-revised filter are further broken down into review articles, journal articles, and clinical studies. (**B**) Year-based distribution of the total number of published articles on intracellular Ca^2+^ in cancer differentiated per the search filters. The yearly distribution of the number of published articles identified using subset-revised filter is uniformly correlated with that of the revised filter; (**C**) Categorization of the number of published articles found using the revised filter per the main intracellular Ca^2+^ signaling molecule(s) in each study. (**D**) Categorization of the number of published articles found using the sub-set revised filter per the main intracellular Ca^2+^ signaling molecule(s) in each study. The articles were sorted by using the categories listed on the y-axis in panels (**C**,**D**) as keywords to be identified in the list of articles (title, abstract, and MeSH words) extracted with Revised Filter 1 by creating a dynamic search feature in Excel.

Intracellular Ca^2+^ signaling involves a smorgasbord of proteins that chelate or transport Ca^2+^ ions across various cellular compartments and thereby assist in signal induction, relay, or integration. With Ca^2+^ signaling being pivotal for vital cellular processes, distortion of this machinery can be highly advantageous to cancer cells. Although there has been a steady upward trend in the number of published studies that delineate the functional exploitation of various intracellular Ca^2+^ signaling components in tumors, limited headway has occurred in translating those research outcomes into clinical applications. To demonstrate this, we first utilized the PubMed database to get a list of all the publications within the realm of intracellular Ca^2+^ in cancer. Using the advanced search feature, we selected “intracellular Ca^2+^” and “cancer” as the keywords to look for in all fields (author, title, abstract, MeSH words, journal name, etc.) of PubMed articles. We got more than 11,000 studies (not shown). As the published articles between 1960 and 1990 only accounted for less than 0.1 percent of the total, we set our year range for subsequent searches as 1990–2020. To minimize the occurrence of non-relevant articles in our dataset (that is, studies only focused on one of the two keywords and not both), we used stricter filtration criteria [Figure 10A]. Based on the distribution pattern of articles by the year and the overall categorization per major intracellular Ca^2+^ molecules, Revised Filter 2 was determined to be a sub-set of Revised Filter 1 [Figure 10B–D]. A notable difference between the two filters in terms of sorting of articles by the intracellular Ca^2+^ signaling molecules in focus was that Revised Filter 2 had a lower percentage of articles on “CaSR or Vitamin D” amongst all the ones with main Ca^2+^ signaling molecules from the endoplasmic reticulum or its interorganellar junctions [Figure 10C,D; from “Calmodulin” up to “Other ER” categories] in Revised Filter 2 (6% (30/454)) than Revised Filter 1 (19% (394/2061)). Therefore, it seemed that the search criteria used in Revised Filter 1 captured most studies relevant to our search without being too stringent; we chose to use the list of articles generated from Revised Filter 1 for subsequent analysis.

**Figure 11:**
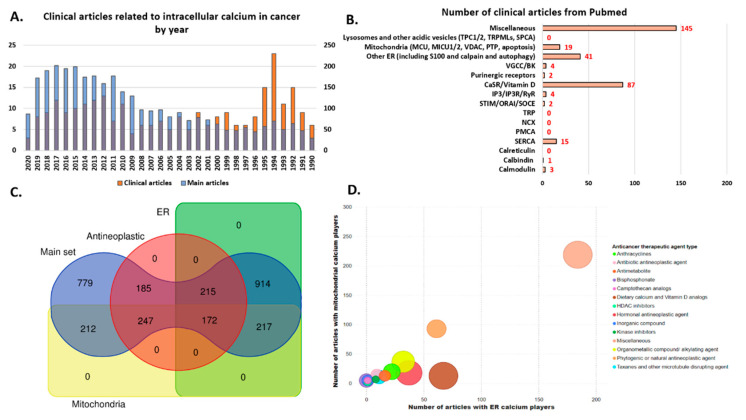
The focus of published clinical studies in the area of intracellular Ca^2+^ in cancer. (**A**) Year-wise distribution of clinical articles (same as clinical studies in the previous figure) extracted from the published articles obtained using the revised filter. The left y-axis corresponds to clinical articles while the right y-axis is for depicting the number of main articles. (**B**) The number of extracted clinical articles categorized by the major intracellular Ca^2+^ signaling molecule(s) each study is focused on. (**C**) The published articles (referred to as the main set) found using the revised filter are broken down into subsets of studies that have utilized antineoplastic agents or highlighted intracellular Ca^2+^ signaling molecules either from the endoplasmic reticulum and its inter-organellar junctions or mitochondria. Venn diagram shows the overlap between the number of studies in each subset and the main set. (**D**) Bubble chart indicates the major groups of antineoplastic agents used in the clinical articles with the main intracellular signaling molecules from the endoplasmic reticulum and its inter-organellar junctions or mitochondria. The size of bubbles is proportional to the number of clinical studies documented in PubMed per anticancer therapeutic agent category.

Clinical studies that were extracted from the list of articles in Revised Filter 1 were labeled in PubMed with one or more of these terms— Clinical Study, Clinical Trial, Clinical Trial Phase I, Clinical Trial Phase II, Clinical Trial Phase III, Clinical Trial Phase IV, Comparative Study, Controlled Clinical Trial, Meta-Analysis, Multi-Center Study, Randomized Controlled Trial, Systematic Review. Two-hundred-and-seventy-seven clinical studies were identified with most of them concentrated between year ranges 1991 to 1996 and 2010 to 2017 (with approximately 10 or more studies published each year) [Figure 11A]. With only 0.1% (277 out of 2941) of the clinical studies for over 30 years, it is interesting to observe that most of such research has explored the role of dietary Ca^2+^ and Vitamin D on cancer (31%) [Figure 11B]. Translational research on any other key Ca^2+^ signaling player in the endoplasmic reticulum or its interorganellar junctions accounted for less than 10 percent of all the published clinical articles. We further evaluated from the main set of published articles the number of studies that used antineoplastic agents (mainly chemotherapeutics) by searching for appropriate keywords. There are 819 articles in the main set that had utilized antineoplastic agent(s) to address a hypothesis related to the role of a major Ca^2+^ signaling molecule in cancer [Figure 11C]. Out of those studies, there are 215 ER-related and 247 that have mitochondrial Ca^2+^ signaling molecules as major components of research. Studies with bisphosphonates and not chemotherapeutics were predominant in the antineoplastic group of articles followed by the ones that utilized dietary Ca^2+^ and/or Vitamin D [Figure 11D]. Among the remaining articles, hormonal, phytogenic or natural, and organometallic agents were the most popular anticancer chemotherapeutics to be used. The above conclusions have been derived with the caveat that PubMed is not an exhaustive database of biomedical research. It is nevertheless surprising to note the lack of clinical development of anticancer therapeutics targeting Ca^2+^ signaling.

**Figure 12:**
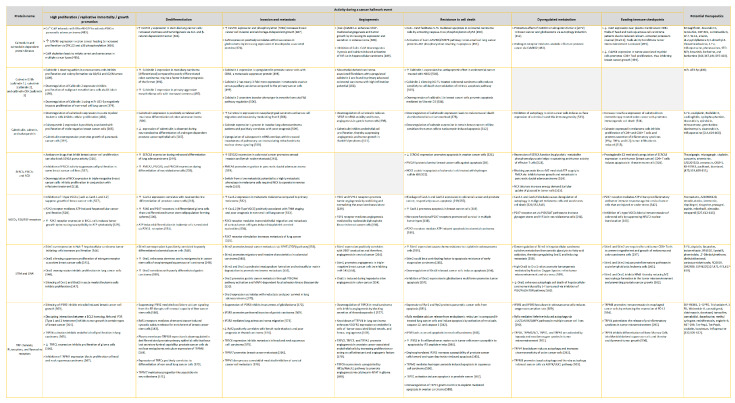

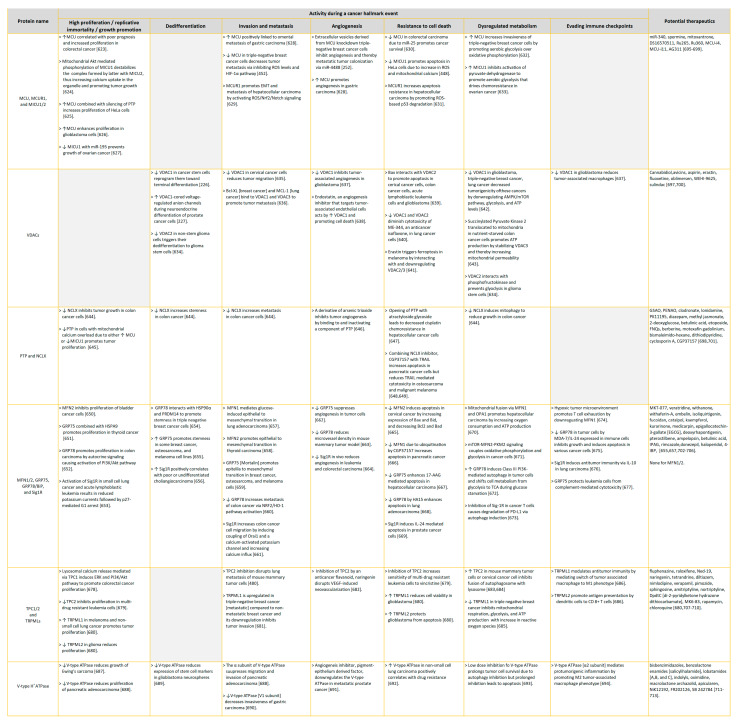
Roles of intracellular calcium regulators in cancer and potential therapeutics.

Research using small molecule drugs targeting numerous Ca^2+^ signaling proteins is currently in the early stages. Some of the examples include RP4010 (ORAI1 inhibitor; Phase I/IB clinical study terminated), Synta66 (SOCE inhibitor; preclinical), CAD204520 (SERCA inhibitor; preclinical), and SKF96365 (TRP channel and SOCE inhibition; preclinical) [471,472,473,474,475,476]. The time-intensive process (median time 7.3 years) of drug development that simultaneously demands heavy intellectual and monetary investments (roughly $648 million per drug in R&D) could be a possible reason for this gap [477]. Toxicity associated with targeting ubiquitously expressed proteins, the heterogeneity within populations of cancer cells, and the development of multidrug resistance are other critical barriers to be overcome [478,479,480,481]. The effectiveness of small molecule inhibitors of Ca^2+^ signaling could be increased by leveraging the advancements made in the nanocarrier-based targeted drug delivery systems. For example, taking from the design of Antp-LP4 related peptides (VDAC1 inhibitors), Venetoclax (a Bcl2 inhibitor) can be encapsulated in inorganic (such as gold nanoparticle or quantum dots) or organic nanocarriers (such as liposomes) coated with transferrin to specifically target cancer cells that demonstrate significant surface expression of transferrin receptors [445,482,483]. However, some studies have also highlighted the independent effect of nanoparticles themselves on the regulation of Ca^2+^ signaling [479]. Drug repurposing is another way to find effective therapeutic modalities targeting Ca^2+^ signaling in cancer. Several FDA-approved anticancer agents (such as 5-fluorouracil, cisplatin, tamoxifen, paclitaxel, and doxorubicin) and drugs against other disease conditions (such as leflunomide, tolvaptan, and teriflunomide) can impact Ca^2+^ signaling machinery [484,485]. Some of these drugs, formulated as prodrugs or nanocarrier loads, are under investigation as antagonists of cancer-promoting Ca^2+^ signaling See Figure 12 [46,226,227,252,440,441,486,487,488,489,490,491,492,493,494,495,496,497,498,499,500,501,502,503,504,505,506,507,508,509,510,511,512,513,514,515,516,517,518,519,520,521,522,523,524,525,526,527,528,529,530,531,532,533,534,535,536,537,538,539,540,541,542,543,544,545,546,547,548,549,550,551,552,553,554,555,556,557,558,559,560,561,562,563,564,565,566,567,568,569,570,571,572,573,574,575,576,577,578,579,580,581,582,583,584,585,586,587,588,589,590,591,592,593,594,595,596,597,598,599,600,601,602,603,604,605,606,607,608,609,610,611,612,613,614,615,616,617,618,619,620,621,622,623,624,625,626,627,628,629,630,631,632,633,634,635,636,637,638,639,640,641,642,643,644,645,646,647,648,649,650,651,652,653,654,655,656,657,658,659,660,661,662,663,664,665,666,667,668,669,670,671,672,673,674,675,676,677,678,679,680,681,682,683,684,685,686,687,688,689,690,691,692,693,694,695,696,697,698,699,700,701,702,703,704,705,706,707,708,709,710,711,712,713].

Even after decades of research on intracellular Ca^2+^ in tumor cells, the vast clinical potential of targeting key players in this area remains underappreciated. Whether it is through redesigning existing therapeutics or developing novel treatments for directly modulating the intracellular Ca^2+^ levels, greater efforts are needed to find ways to reverse the hijack of intracellular Ca^2+^ signaling in cancer.

## Figures and Tables

**Table 1:** Sarcoplasmic or endoplasmic Ca^2+^-ATPase isoforms.

SERCA Isoform	Tissue Localization	Inhibitor(s)
SERCA1	Adult and neonatal skeletal muscles	Myoregulin (MLN), PLN, and SLN
SERCA2a	Cardiac muscles	PLN, SLN, miRNA-25
SERCA2b	Non-muscle tissues	ALN, TM11 and its luminal extension
SERCA3	Co-expressed with SERCA2b in endothelial, epithelial, and hematopoietic cells.	Endoregulin (ELN)

SERCA has three isoforms and multiple splice variants. The table summarizes the localization of major isoforms and splice variants along with a mention of their endogenous inhibitors [29].

**Table 2:** Protein interactions of TRP channel isoforms.

TRP Isoform	Protein Interactors
TRP A	PIP_2_, AKAP 79/150, Ca^2+^-CaM, TRPV1, Sig-1R
TRP C	CaBP1, CaM, Caveolin1, Homer, Immunophilins, IP_3_Rs, Junctate, Junctophilin, NCX1, PLCγ1 RhoA, Stathmin, VAMP, ZO-1, actin cytoskeleton (myosin, ERM proteins, NHERF, etc.), focal adhesion kinase, contactin, Src, STIM, ORAI, RyR, TRPV4, TRPP1, TRPM4
TRP M	14-3-3γ, 5HT1B, AKAP5/150, CaM, PKCα, PTPL1, Rac1, S100A10, Sig1R, TRPC3, RACK1, ENAC, Synaptotagmin1, α-actin, Myosin heavy chain, Annexin 1, G_αq_
TRP ML	PDCD6, Cdc42, HSP40, HSP90, Rho1, Rac1, Rac2, RhoG, TPC1, TPC2, TRPV5
TRP P	EGFR, eIF-2α, Filamin-A, HDAC6, IP_3_R1, IP_3_R3, PERK, α-actinin, PKD1, PKD1L1, RACK1, PLCγ, Troponin I, Tropomyosin, TRPC (1–4), TRPV4, RyR2,
TRP V	AKAP5/150, Calbindin D-28k, CaM, Caveolin1, Cyclophilin B, CAMKII, E-cadherin, EGFR, e-NOS, GABARAP, Fyn, F-actin, IP_3_R3, Klotho, Lck, Lyn, Myosin, α-integrin, α-tubulin, PKC, PPARα, S100A10, Src, TMEM16A, TRPA1, TRPC1, TRPML3, TRPP1

The table provides a list of major protein interactors recognized for each TRP isoform [58,63,64,65,66,67,68].

## Data Availability

Not applicable.
